# Selenium Nanoparticles: Green Synthesis and Biomedical Application

**DOI:** 10.3390/molecules28248125

**Published:** 2023-12-15

**Authors:** Ekaterina O. Mikhailova

**Affiliations:** Institute of Innovation Management, Kazan National Research Technological University, K. Marx Street 68, 420015 Kazan, Russia; katyushka.glukhova@gmail.com

**Keywords:** selenium nanoparticles, green synthesis, capping agents, antibacterial activity, anticancer activity

## Abstract

Selenium nanoparticles (SeNPs) are extremely popular objects in nanotechnology. “Green” synthesis has special advantages due to the growing necessity for environmentally friendly, non-toxic, and low-cost methods. This review considers the biosynthesis mechanism of bacteria, fungi, algae, and plants, including the role of various biological substances in the processes of reducing selenium compounds to SeNPs and their further packaging. Modern information and approaches to the possible biomedical use of selenium nanoparticles are presented: antimicrobial, antiviral, anticancer, antioxidant, anti-inflammatory, and other properties, as well as the mechanisms of these processes, that have important potential therapeutic value.

## 1. Introduction

Green technologies are a relatively young trend developed in recent decades with interest growing like a snowball. This is not surprising, because the goal pursued in their development is to protect nature, eliminate the damage caused to the environment in the past, and preserve the Earth’s natural resources. Therefore, searching for ways to synthesize various compounds using eco-friendly technologies is the most important task. Nanoparticles have become one such topic deserving close attention. Their huge variety (inorganic nanoparticles, organic nanoparticles, ceramic nanoparticles, and carbon-based nanoparticles [1,2,3,4,5,6]), biosynthesis simplicity, and remarkable physicochemical, biological, and catalytic properties can literally open a new era for the creation of modern drugs solving different biomedical tasks. The biological synthesis of nanoparticles by living organisms—bacteria, fungi, and plants [7]—is an inexhaustible source of medicines that enable the solving of large-scale problems in the treatment of various diseases—bacterial and viral infections, oncological diseases, parasitic invasions, inflammatory processes, diabetes, and also in biosensing, bioimaging, drug delivery, diagnostics, etc. [8,9,10].

Metal nanoparticles are one of the most popular objects in green synthesis, and the number of articles on this subject is steadily growing from year to year. Biosynthesized silver nanoparticles with a wide range of useful biological properties are the most popular among them [11,12]. However, other nanoparticles are of particular interest. Among those are selenium nanoparticles. The selenium (Se) element has the atomic number 34 and is located in group 16 and period 4 of the D.I. Mendeleev chemical element periodic system. Selenium is a non-metal, and it is classified as a chalcogen element. It was discovered by J.J. Berzelius in 1817 during his search for the method of sulfuric acid production [13]. Selenium received its name (from Greek σελήνη—the Moon) due to the fact that in nature it is a satellite of chemically similar tellurium (named after the Latin word telluris—Earth). For a long time, until the middle of the 20th century, selenium was considered poisonous, but in 1957, an important discovery inverted this idea upside down—it turned out to be able to prevent severe myodystrophy and cirrhosis of the animal liver [14]. Moreover, in the 1970s, it was found that selenium is part of proteins containing amino acids such as selenmethionine, selencysteine, and methylselenysteinine [15,16]. Selenium’s vitality for humans and animals was shown in further studies. Selenium as a trace element plays an important role in various physiological process: it is necessary for normal immune system functioning, participates in redox process regulation and iodine metabolism, and also performs anti-inflammatory and antiviral functions [17,18,19]. Selenium also acts as an antioxidant enzyme cofactor (glutathione peroxidase and thioredoxin reductase) protecting the human body from reactive oxygen species (ROS) [20]. Recently, the potential role of selenium in reproductive function in women and fertility in men was revealed [21,22]. There is a lot of evidence in favor of Se as an inducer of cancer cell apoptosis with minimal side effects on normal cells [23]. In 2009, the WHO even provided data on the daily intake of selenium, which should be 50–55 µg per day in the human diet, depending on individual body weight [24]. Selenium deficiency can lead to serious disorders in the body—liver necrosis, muscular dystrophy, and thyroid dysfunction [25]—and can also cause disorders in the heart, bones, muscles, etc. [26], while normal Se consumption contributes to growth and fertility improvement (shown in domestic chickens) [27]. Unfortunately, the line between selenium deficiency and toxicity is very thin, so it is extremely important to take this into account when creating drugs based on it. Selenium compounds exist in nature in four oxidation degrees: selenate (Se^6+^), selenite (Se^4+^), selenide (Se^2−^), and elementary selenium (Se^0^) [28]. The first three forms are toxic even at low concentrations, and only the latter is insoluble in water and, in essence, is non-toxic [29]. To achieve the desired therapeutic effect, selenium-based nanoparticles can be created, which can alleviate toxicity problems. In this regard, SeNP synthesis mediated by living organisms (bacteria, fungi, algae, and higher plants) can solve several important problems at once: to be an environmentally friendly alternative to expensive, energy-intensive, and potentially toxic chemical and physical methods for producing nanoparticles and to create nanoparticles with diverse and unique biological properties by the virtue of “green” bioreactors. Biocompounds can not only take part in nanoparticle synthesis but also act as capping agents capable of preventing agglomeration, provide functional groups for the drug attachment [30], stabilize NPs and, due to their own biological potential, have a targeted effect. This review is devoted to selenium nanoparticle biosynthesis, as well as their effect mechanisms on living organism cells and their potential biomedical applications.

## 2. The Proposed Mechanism of SeNPs

The creation of nanoparticles using biological methods has provoked a real boom in this nanotechnology field over the past decades. Environmentally friendly green synthesis, which does not require high energy costs or the use of hazardous and toxic substances, has other undeniable advantages compared to physical and chemical methods for producing nanoparticles. The application of biological substrates (bacteria, fungi, algae, and plants) as a matrix for the NP synthesis and packaging allows for the production of an immeasurable number of variants, each having their own, sometimes unique properties, provided by nature itself and the organism where the process takes place. Low toxicity, biodegradability, and biocompatibility are the key parameters of the bionanoparticle’s positive reputation. Metal nanoparticles synthesized biologically are extremely popular objects [31,32,33]. Among non-metallic NPs, special attention is being focused on selenium nanoparticles. The interest in selenium compounds is not an accident as they directly participate in the most important biological processes in human and animal cells. Selenium compounds in the Se^0^ form are the least toxic, which makes them a potential aid in the fight against various diseases.

**Figure 1:**
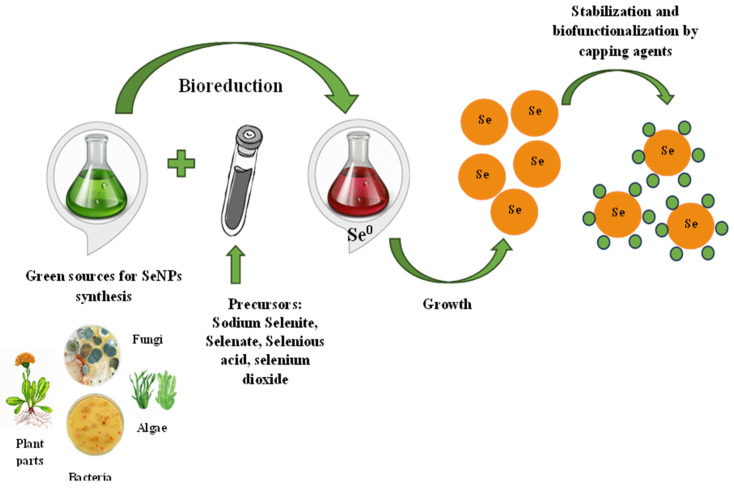
The proposed mechanism of selenium nanoparticles synthesis.

SeNP biosynthesis is a simple, one-step process that does not require toxic chemicals, high temperatures, or complex equipment. It is possible to realize green synthesis in two ways—directly in living organisms or with the help of bioreagents extracted from them. Sodium selenite/selenate, selenous acid, and selenium dioxide can be used as precursors for the synthesis of SeNPs added to a “bioreactor”—bacteria or fungi culture fluid, plant extract, etc. [34,35,36]. The precursors’ addition to the bioextract leads to the appearance of a red, red–orange, or orange solution color, indicating the reduction of selenium compounds and the formation of colloidal SeNPs. Further, the processes of the stabilization and capping of nanoparticles take place [37]. The proposed synthesis mechanism is presented in Figure 1. The selenium salt reduction to Se^0^ occurs due to different biopolymers capable of performing this role. For example, it is assumed that the enzymes thioredoxin reductase, nitrite reductase, or other membrane reductases are responsible for SeNP synthesis in bacteria [38]. Bacterial reductases have the ability to reduce the water-soluble oxyanion SeO_4_^2−^ to SeO_3_^2−^ and then to insoluble elemental selenium Se^0^ in a two-stage reaction. A variety of proteins, terpenoids, vitamins, flavonoids, tannins, polysaccharides, and other biologically active substances may be responsible for both the reduction process and SeNP stabilization and capping, preventing their agglomeration in aqueous solution during plant synthesis [39]. Capping agents, as important nanoparticle components, are very attractive to study owing to their own therapeutic significance. Frequently having biological functionality, these biocompounds are able to enhance the SeNPs’ medical effect and reduce their toxicity. This is especially true for plant SeNPs, because medicinal herbs from all over the world, long known to mankind due to their beneficial effects on the body, are used for their production. Selenium nanoparticle formation and their shape, size, and capping agent composition are influenced by factors such as the precursor and its concentration, temperature, pH, and of course, the “factory” where the biosynthesis process takes place [40,41,42].

Transmission electron microscopy (TEM) and scanning electron microscopy (SEM), used to estimate the size and shape of SeNPs, can be distinguished as widely used methods for assessing the biosynthesized selenium nanoparticle properties: transmission electron microscopy (TEM), UV/Vis spectrophotometry, and dynamic light scattering (DLS) to evaluate the nanoparticle physical properties; scanning electron microscopy (SEM) to analyze the morphology of the nanoparticles; X-ray diffraction measurements to study the nanoparticle structure; as well as FTIR analysis (Fourier transform infrared spectroscopy), providing the opportunity to characterize biomolecules involved in SeNP synthesis and stabilization [43,44,45].

### 2.1. By Bacteria

Biologically active substance synthesis by microorganisms is a very popular method that has become increasingly important in recent years. This is a simple, cost-effective, environmentally friendly method widely used in biotechnology. SeNP biosynthesis is no exception, especially due to bacteria being capable of the biotransformation of selenium compounds. Apparently, this is one of the leading reasons for the large-scale microorganism-mediated SeNP production, in contrast to metal nanoparticles, where plant nanoparticles occupy the lion’s share of research [46]. The used microbes are extremely diverse—from bacteria of the genus *Bacillus*, *Pseudomonas*, *Lactobacillus*, and *Escherichia coli* to more exotic microorganisms.

Literature data indicate that both Gram-positive and Gram-negative bacteria can synthesize SeNPs. The reduction of SeO_3_^2−^ and SeO_4_^2−^ to Se^0^ is one of the mechanisms for toxic Se oxyanion removal in aerobic and anaerobic conditions [47]. Bacteria are able to implement this process intracellularly (in the periplasmic space) and extracellularly; however, the extracellular variant seems to be more preferable because nanoparticles are easier to extract. Data on some of them are presented in Table 1. The intracellular mechanism consists of several stages: the Se oxyanions transport into the cell mainly due to sulfate permeases; these compounds’ reduction by bacterial enzymes to Se^0^ proceeds with their subsequent release from the cell; the elementary Se^0^ assembling into SeNPs by the continuous reduction of Se oxyanions to Se^0^; and the isolation of SeNPs from bacterial cells using centrifugation and filtration methods. Thus, the synthesis of selenium nanoparticles was intracellularly carried out by Gram-positive bacteria *Bacillus cereus* in two consecutive steps from selenate to selenite and then elemental selenium [48]. The synthesis of SeNPs by halophilic bacteria *Halomonas eurihalina*, as well as for *Klebsiella pneumoniae*, was found to be intracellular [49,50].

An interesting object for intracellular selenium nanoparticle production are the Gram-negative bacteria *Delftia* sp., known for their unique metabolic abilities to transform various pollutants; in this case, the reduction of selenite to SeNPs did not have harmful effects on the cell structure [51]. Moreover, selenium nanoparticles synthesized intracellularly using *Lactobacillus pentosus* were agglomerated in the surrounding medium [52]. Khoei et al. showed that after the synthesis by *Burkholderia fungorum*, SeNPs were present both intracellularly and extracellularly. It is assumed that the SeNP formation mechanism can be conditionally attributed to cytoplasmic enzymatic activation mediated by electron donors. Then, biogenic nanoparticles are probably released from bacterial cells as a result of cell secretion or lysis [53]. The following hypotheses of SeNP export from the cell can be found in the literature: (1) the bubbles’ formation on the outer membrane (membrane vesicles), occurring in response to stress by encapsulating various harmful substances in the bubbles of a lipid bilayer membrane for removal outside the cell; (2) secretion due to the specialized proteins (for example, the SefA protein in *Thauera elenatis*) [53,54]. Extracellular SeNP synthesis was shown for *Pseudomonas alcaligenes* [55], *Enterococcus faecalis* [56], *Bacillus subtilis* [57], on the membrane surface of bacteria isolated from wastewater from glass factories [58], etc. Such synthesis is preferable to intracellular because it does not require further isolation of nanoparticles from the cell.

Despite the mechanism of bacteria-mediated biosynthesis not being fully understood, the most important role is assigned to enzymes. It was found that NADH-dependent reductases are responsible for the biomimetic reduction of SeO_3_^2−^ to Se^0^ nanospheres in *Pseudomonas aeruginosa* [43]. Nitrate reductases are capable of reducing in *E. coli* [59] and arsenate reductase in *Bacillus selenitireducens* [60]. It is proposed that membrane-bound reductases are able to produce reduced Se^0^ through enzymatic electron transfer [61]. Thus, in *B. fungorum* strains synthesizing SeNPs, cytoplasmic reductase accepts electrons from NADH and NADPH. Free thiols and thiol groups of proteins may participate in SeO_3_^2−^ reduction in both strains as well [53]. In addition, these enzymes can also act as stabilizing agents. Selenium nanoparticle synthesis in *Proteus mirabilis* was observed in the cell membrane system due to the reductase catalytic activity, while NADH/NADPH are electron donors, so SeNP synthesis occurred inside the cell with subsequent release outside [62].

However, there is evidence of another mechanism of SeNP bacterial synthesis. In the filamentous bacterium *Streptomyces* sp., the intracellular Se(IV) reduction was usually driven by reduced thiols such as glutathione (GSH) in the cytoplasm via the Painter reaction, and further, SeNPs were released through cell lysis or fragmentation [63]. The mechanism suggests that selenite is converted by glutathione or glutathione reductase as the main electron donors for hydrogen selenide (H_2_Se) via selenodiglutathione (GS-Se-SG) and glutathionylselenol intermediates. Initially unstable selenodiglutathione formed, and then reacts with residual GSH to form diglutathione (GSSG) and elemental selenium. The electron source for GSH regeneration is mainly NADPH. This variant of SeNP synthesis involving GSH was discovered for *Idiomarina* sp. [64]. Glutathione reductase and selenocysteine lyase can participate in the selenium reduction to SeNPs in lactobacilli [65]. Sulfite reductase and thioredoxin reductase, located in the Alcaligenes faecalis cytoplasm, apparently participate in selenite reduction and NP synthesis in the presence of NADPH or NADH as electron donors [66]. Thioredoxin reductase (TrxR) in *B. subtilis* can use NADPH as an electron donor for direct selenite reduction to selenium nanoparticles [54].

The bacterial synthesis of SeNPs is directly related to the microorganism’s growth phase. Some researchers testify to the maximum SeNP synthesis in the logarithmic growth phase, confirmed by the optimal concentrations of reducing agents in this phase [53,54,67]. Selenium nanoparticle synthesis in *Azospirillum brasilense* was observed in the late logarithmic phase [68]. Similar data were found for *B. fungorum*, where SeNPs were produced intracellularly and localized mainly extracellularly [53]. However, the greatest effect was achieved in the stationary phase, as, for example, in *Azoarcus* sp. [69]. It was suggested that when cells were highly energized, i.e., at the exponential growth phase, selenite could be secreted out of the cell. However, when the energy potential is depleted in the stationary phase, selenite is able to persist in the cytoplasm for a sufficient time and turn into SeNPs. Selenium nanoparticle synthesis at the beginning of the stationary phase also involves many lactobacilli [70]. Additionally, the cellular reductase production and consumption involved in selenite reduction are mediated by the bacteria’s growth phase.

Interestingly, *Azoarcus* sp.-mediated SeNPs can be produced both in anaerobic and aerobic conditions [69]. Analogous data were obtained for SeNP synthesis using selenite-reducing bacteria, *Citrobacter freundii*, and the anaerobic selenite reduction was more rapid and pronounced than under aerobic conditions [71]. The synthesis conditions (aerobic or anaerobic) leave their mark on its features. For example, in *Paenibacillus terreus*, Se oxyanions act as electron acceptors and form extracellular granules composed of stable, uniform nanospheres of SeNPs having trigonal structures, or the selenite is reduced by a Painter-type reaction in which Se-digluthathione intermediates are formed followed by SeNPs (because selenite is highly reactive with thiol groups) [72].

The size and shape of nanoparticles are significant NP characteristics, playing an important role in biological functions. The size varies over a wide range and depends on the “biofactory” used for the selenium nanoparticle synthesis. The shape is most often spherical or sometimes amorphous, as, for example, for SeNPs from *Lactobacillus paralimentarius* [73]. The size of SeNPs synthesized by *Pantoea agglomerans* depended on the incubation time, demonstrating a direct relationship between the incubation time and the nanoparticle size: an increase in the incubation time increased the detected SeNPs’ size [61]. Wang et al. also confirmed this hypothesis: after aging for 24 h, the particles had already grown to about 50–150 nm, and there was evidence of the final spherical structure. After aging for 48 h, a very large percentage of the nanoparticles had grown, with diameters from 50 to 400 nm [74].

The transformation process of small precursor nanoparticles into large ones corresponded to the typical Ostwald maturation process. The same nanoparticle maturation was described for *Ralstonia eutropha* with nanoparticle sizes ranging from 40 to 120 nm [75]. However, nanoparticles with a smaller diameter are of the greatest interest for further biomedical use because SeNPs with a particle size of less than 200 nm easily penetrate into cells, participate in metabolic processes, and, thus, exhibit higher biological activity [57].

**Table 1:** Bacteria-mediated SeNPs.

Species	Size, nm	Shape	Synthesis	Reducing and Capping Agents	Reference
*Pseudomonas aeruginosa*	~21	spherical	extracellular	enzyme	[40]
*B. cereus*	~170	hexagonal structures without amorphous shapes	intracellular		[45]
*Halomonas eurihalina*	~260	spherical	intracellular	proteins and polysaccharides	[46]
*Delftia* sp.	~192	spherical	intracellular	proteins and carbohydrates	[48]
*Lactobacillus pentosus*	~106	spherical	intracellular	alcohol, aldehyde, aliphatic amine, and aryl disulfides	[49]
*Burkholderia fungorum*	170–200	spherical	intracellular	proteins	[50]
*Enterococcus faecalis*	29–195	spherical	extracellular	-	[54]
*B. subtilis*	126	spherical	extracellular	-	[55]
*Pantoea agglomerans*	~100	amorphous	intracellular	proteins	[58]
*Providencia vermicola*	3–50	hexagonal monodispersed	intracellular	proteins	[59]
*Idiomarina* sp.	150–350	spherical	intracellular	thiols, including the non-protein thiols (NP-SH)	[61]
*Alcaligenes faecalis*	273	spherical	extracellular	proteinsand carbohydrate residues	[63]
*Lactobacillus acidophilus*	2–15	spherical	extracellular	proteins	[64]
*Azoarcus* sp.	123	spherical	intracellular	-	[66]
*B. subtilis*	5–400	spherical	extracellular	proteins	[71]
*Ralstonia eutropha*	40–120	spherical	extracellular	proteins and enzymes	[72]
*Lactobacillus casei*	50–80	spherical	intracellular	proteins and polysaccharides	[75]

The initial selenite concentration for the synthesis is also important: an increase in the sodium selenite concentration in the medium led to the formation of small amounts of bacterial SeNPs [57].

Capping agents are one of the most fundamental nanoparticle components. Playing a critical role in NP stabilization and preventing nanoparticle aggregation, they can make a considerable contribution to the biological properties of selenium nanoparticles. Surface nanoparticle modifications due to capping agents make them stable, and their suspensions are well dispersed, since they act as coating agents. Proteins, enzymes, polysaccharides, and lipids often work as capping agents in bacterial SeNPs. For instance, in *Providencia vermicola*-mediated SeNPs, surface-bound proteins act as natural capping agents, preventing agglomeration and exhibiting promising medical activity [76]. This interaction can be realized due to the electrostatic interaction between selenium atoms and the NH and C=O groups; in addition, the interaction can also be carried out with a variety of high-affinity proteins [77]. It is assumed that the long-term solution stability of the SeNPs from *B. subtilis* caused by the presence of proteins as capping agents binding to the nanoparticle surface and preventing their aggregation [74].

In addition, it is believed that proteins and polysaccharides covering nanoparticles can reduce the toxicity of *L. casei* SeNPs [78]. SeNPs created by *Bacillus paralicheniformis* were capped with exopolysaccharides, enhancing their antioxidant properties to remove free radicals and protect cells from oxidative stress caused by hydrogen peroxide [79]. Amides, aromatic amines [80], alcohols, phenols, carboxylic acid, aromatic compounds, alkanes, alkenes, and fatty acids [81], as well as peptides and aldehydes in nanoparticles from *Streptomyces* sp. [82] can also act as capping agents.

### 2.2. By Fungi

Unfortunately, there are few data on the mechanism of SeNP synthesis by fungi, although research is underway. Fungi-mediated SeNP synthesis is possible both intracellularly and extracellularly. Fungi as nanoparticle manufacturers attract attention owing to their ability to secrete many extracellular metabolites that can increase the nanoparticle yield and their stability. Thus, extracellular selenium nanoparticle synthesis was presented in *Penicillium crustosum* as a producer of various enzymes (amylase, cellulase, gelatinase, and xylanase) [83]. At the same time, SeNPs in *Mariannaea* sp. were biosynthesized intracellularly, released from cells, and subsequently increased in size outside the fungal cell [84]. It is supposed that the key role in the synthesis process belongs to enzymes working as reducing and stabilizing agents. Moreover, a lot of proteins are adsorbed on the surface of nanoparticles, forming a stable selenium bioconjugate system, and lipid and amide groups might play an important role during the formation process [84]. The intracellular synthesis mechanism is characteristic of *Saccharomyces cerevisiae* [85]. There are suggestions that membrane-bound oxidoreductases and quinones were engaged in metal NP synthesis using yeast strains. The possible involvement of sulfite reductase in nanoparticle formation in *Saccharomyces boulardii* was shown [86]. As the pH value increased in the yeast’s internal environment, reductase-reducing metal ions with the simultaneous production of NPs were activated. Quinones also have strong nucleophilic and redox properties, making them more applicable for metal ion reduction and their conversion into NPs. Tugarova et al. suggest that in *Azospirillum*, selenite reduction is under the action of either nitrite or nitrate reductase localized in the cell. This reduction process is not just a protective mechanism for detoxifying selenite to a less toxic, insoluble Se, but it may be due to this toxic compound’s inclusion in the nitrogen metabolic cycle while its transformation occurs under the action of the enzyme(s) responsible for denitrification [87]. In another synthesis option, selenite can be reduced to SeNPs in *Penicillium citrinum* as a result of a reaction with the reactive thiol groups of the protein/peptide—using glutathione reductase [88].

High pH values of the medium had a positive effect on selenium nanoparticle synthesis [83,84,87,89]. Also, the alkaline medium prevented NP agglomeration and stabilized the coating agents on the NP surfaces by interacting with protein amino groups [84]. As in the case of bacteria, the microorganism’s growth phase is important. So, the stationary growth phase for maximum *Trichoderma* sp. in SeNP synthesis was demonstrated [89].

Capping agents are striking parameter-stabilizing nanoparticles and are of particular interest for their further biomedical use. As such, proteins and enzymes can bind to the formed SeNPs in several ways—by cysteine residues, free amino groups, and the electrostatic attraction of negatively charged carboxylate groups [83], as well as lipids [84], alkenes, alkanes, alcohols [89], polysaccharides [90], phenols, primary amines [91], carbohydrates, and amino acids [92].

### 2.3. By Algae

Another original “bionanofactory” for the production of selenium nanoparticles is algae. With many valuable biologically active compounds capable of selenium reduction and participating in nanoparticle coating, algae can be an effective tool for creating SeNPs. Such synthesis can be mediated by a variety of compounds, such as amines, amides, alkaloids, terpenoids, antioxidants (polyphenols and tocopherols), polysaccharides, proteins, pigments (carotenoids), and phycobilins contained in algae extracts and involved in selenium reduction and nanoparticle stabilization [93]. However, this mechanism is practically unstudied. Thus, synthesis in cyanobacteria (blue–green algae) was found both extracellularly, or on the surface of the cell wall, and inside the cell [94]. Selenate reductases and selenite reductases are responsible for intracellular synthesis, while extracellular synthesis occurs under the action of various phytocompounds with reducing potential [94].

For example, extracellular synthesis is indicated for *Spirulina platensis* [95], *Arthrospira platensis* [96], and *Anabaena variabilis* [97]. In addition, *Spirulina platensis*-based synthesis proceeded in the exponential growth phase, which can be considered from the standpoint of maintaining cyanobacteria samples in the exponential growth phase as a strategy for the large-scale production of SeNPs with high yield [98]. For brown algae as well as for blue–green algae, for example, for *Polycladida myrica*, alkenes, alcohols, phenolic groups, carbonyl-unsaturated ketone amides, amino acids, esters, and proteins were found in the capping agent composition [99], and for *Sargassum angustifolium*, polysaccharides (such as Fucoidan) [100].

### 2.4. By Plants

The huge diversity of plant selenium nanoparticles is so great and difficult to describe. The rich flora of our planet and the knowledge accumulated about medicinal plants (for example, traditional Chinese medicine or Ayurveda) and their disease treatment application, along with well-known plants consumed in food, allow using different herb parts as a “biofactory”—roots from *Blumea axillaris* [101], peel from *Solanum melongena* [102], wheat seedlings from *Triticum aestivum* [103], leaves from *Azadirachta indica*, *Petroselinum crispum* [104,105], and others, stem from banana [106], flowers from *Calendula officinalis* [107], and many others. However, since the vast majority of research is conducted in order to obtain a targeted effect (antibacterial, anticancer, antioxidant, anti-inflammatory, antiparasitic, etc.), the mechanism of SeNPs plant synthesis is not given such significant attention. Nevertheless, based on the obtained results, a variety of plant biomolecules—proteins, enzymes, flavonoids, terpenoids, sterols, polysaccharides, vitamins, phenolic compounds, organic acids, enzymes, and lignin—take part in nanoparticle synthesis and packaging, also typical for other nanoparticles, including metallic ones [108]. Apparently, this process is specific to each plant. Reducing agents from plant extracts are electron donors for metal ions and convert them into nanoparticles. From this point of view, secondary plant metabolites are very important in green SeNP synthesis; they act as selenium reductants, but also prevent NP aggregation and promote the formation of smaller particles [109].

The smaller size and high ratio of surface area to volume of NPs give them the possibility to interact closely with the microorganism cell membrane, which facilitates interaction and intracellular diffusion, causing significant damage to the membranes and having a toxic effect on DNA or cell proliferation inhibition by processes mediated by reactive oxygen species [107]. For example, in onion and ginger extracts, selenium is reduced by phenolic compounds (quercetin and gingerols), where the enol group of the extract’s phenolic compounds breaks the O-H bond, which releases an electron to reduce Se [110]. Polyphenolic compounds contain different functional groups capable of forming nanoparticles. It is assumed that the tautomeric conversion of a flavonoid from an enol to a keto form can release a chemically active hydrogen atom that can reduce metal ions to form NPs during synthesis using aquatic extracts of black and green tea [109]. Moreover, some flavonoids have the ability to chelate metal ions with their carbonyl groups or electrons. One of these flavonoids with a very strong chelating effect, quercetin, was found in an infusion of green tea. These mechanisms may explain the flavonoid’s ability to adsorb on the NP surface.

It is supposed that they participate not only at the reduction stage but also at the initiation stage of NP formation and their further aggregation [109]. In *Crocus sativus* extract-mediated synthesis, reduction can occur due to catechin and gallic acid. As a rule, the ortho-hydroxyl groups of these compounds are involved in SeNP synthesis. Se^4+^ is reduced to Se^0^ when two electrons are released in the flavonoid ring. The catechin ring is oxidized to a 3,4-quinone ring as a stable final product. During the process, Se undergoes additional aggregation into larger clusters, eventually forming SeNPs. The quinone form of catechin also binds to the NP surface, leading to less aggregation of Se^0^ at the nanoscale [111].

The pH of the medium for SeNP synthesis is also significant, as it affects the bioreduction of precursors. For example, in *Muntigia calabura*-mediated synthesis, it was found that Se nanoparticles retained a spherical shape in the pH range of 5–6, while a neutral medium ensured the joint presence of spherical and nanorod nanoparticles, i.e., the pH level directly affected the formation of nanomaterials, especially during crystal formation [112]. Due to the presence of various compounds in *M. calabura* extract, the surfactant layer can be formed and effectively maintain the spherical nanoparticle shape without any agglomeration. However, an increase in the pH level can change the crystallization process, leading to changes in the nanoparticle shape [112]. For the synthesis of SeNPs using orange peel extract (*Citrus reticulata*) the maximum Se reduction occurred at pH 4 [113]. However, during the *Portulaca oleracea* synthesis, the optimal pH value was 8, indicating that the functional groups present in the aqueous plant extract were more active under alkaline conditions [114]. At the same time, SeNPs synthesized using garlic cloves (*Allium sativum*) in aqueous solutions with different pH values (ranging from 4 to 9) were stable without any aggregation [115]. A pH value in this range is good for the human physiological system, so selenium nanoparticles made from garlic can be used in the future for a wide spectrum of biological applications [115].

Since nanoparticles tend to agglomerate, their stabilization is necessary to suppress their excessive growth by coating them with a polymer or surfactant layer, decreasing the interaction between nanoparticles. Biocompounds can act as phyto-capping agents, including those contributing to the reduction of selenium salts to SeNPs. The selenium nanoparticle’s stability is usually assessed by measuring the zeta potential. SeNPs synthesized by plant extracts are covered with a bio-organic layer, consisting of proteins, polysaccharides, and lipids, with a significant proportion of ionized carboxylic groups. These groups, specific to both the side chains of some amino acid residues and carboxylated polysaccharides, are responsible for the negative values of the SeNP zeta potentials; the higher the negative values of the zeta potentials, the more stable the nanoparticles [116]. In addition, phytocompounds can interact with the selenium precursor and control the growth of particles in three dimensions, giving them a spherical shape [117]. However, the capping agent’s essential role is to create the biological functions of SeNPs according to their own therapeutic potential. Polysaccharides, phenolic chemicals, flavonoids, tannins, saponins, amino acids, enzymes, proteins, and sugars are biomolecules contained in plant extracts that have medical value and act as plant capping agents. For example, tannins can have anti-inflammatory and antioxidant effects, as well as antimicrobial activity, making them “plant antibiotics”; antidiuretic properties were discovered for alkaloids; saponins provide hypocholesterolemic and antisclerotic effects; flavonoids have antioxidant, anti-inflammatory, and antimicrobial functions; terpenoids possess insecticidal properties [102]. Therefore, the covering layer of plant-synthesized selenium nanoparticles is extremely critical from the point of view of their further medical use. SeNP packaging involves diversified functional groups and compounds, such as carboxyl groups [118,119], alcohol groups [120,121], phenolic groups [122], amines [123], amides [124], alkanes [120], aldehydes, ketones [125], esters [126,127], amino acids [114], tannins [123], flavonoids [117,128,129,130], cannabinoids [131], proteins [132,133,134,135], polysaccharides [136,137], ascorbic acid [138], carbohydrates [135,139], polyphenols [140,141], terpenoids [142,143], sugars [144], alkaloids [145,146], glycosides [145], and saponins [146].

### 2.5. Other Biosynthesis

Green selenium nanoparticle synthesis is not limited to different “biofactories”. Separate biomolecules having biological activity are also used, providing anticancer, antioxidant, anti-inflammatory and other impacts. Such examples include SeNP synthesis using berberine, a plant alkaloid with anticancer and antisclerotic effects [147]; apigenin is one of the most common aglycones of flavones, a natural antioxidant with anti-inflammatory and anticarcinogenic properties [148]; bee propolis, known for many centuries for its antimicrobial, antioxidant, anti-inflammatory, immunomodulatory, and cardioprotective properties, can synthesize nanoparticles due to its broad range of biomolecules like phenols, flavonoids, alkaloids, steroids, terpenoids, etc. [149]; by bioactive compounds of honey [150]; by Carvacrol—a member of the monoterpenoid phenols, possessing antimutagenic, analgesic, antimicrobial, antitumor, antiparasitic, and antioxidant activity [151]; by enzyme synthesis using bovine serum albumin as a reducing agent [152]; with naringenin and baicalin participation as capping agents—flavonoids with antimicrobial and anti-inflammatory effects [153]—and zein (a class of prolamine proteins contained in corn) [154]. Such an abundance of approaches to biosynthesis and applied biological objects represent an inexhaustible storehouse of possibilities both for SeNP production and for further biomedical application.

## 3. Green SeNP Application

**Figure 2:**
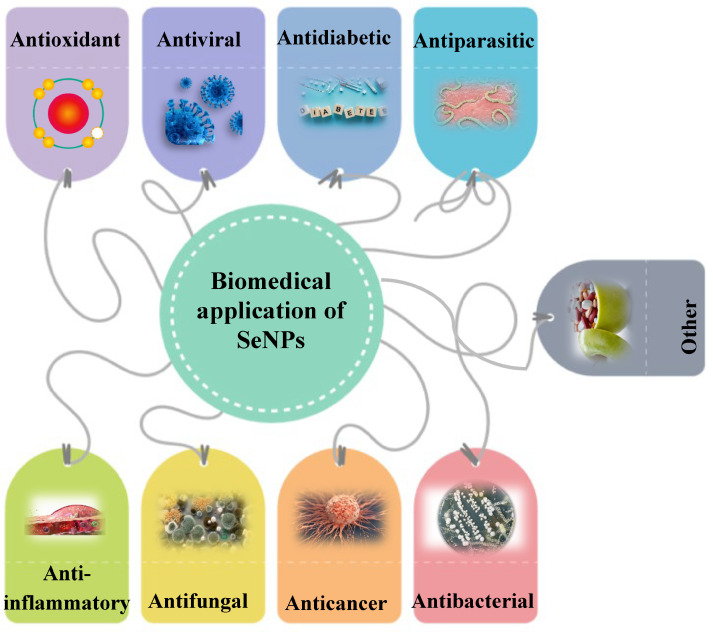
Biomedical application of SeNPs.

SeNPs have great potential for biomedical application according to their remarkable properties (Figure 2). They have many advantages and useful therapeutic properties, which will be discussed below.

### 3.1. Antibacterial Activity

The search for new antibacterial drugs is a very urgent problem due to the increasing resistance of pathogenic microorganisms to well-known and widely used antibiotics. Human pathogens are the most popular for selenium nanoparticle antimicrobial activity studies. These include *Staphylococcus aureus*, which, upon entering the bloodstream, can cause endocarditis, pneumonia, osteomyelitis, and other infections. In addition, hospital strains, such as methicillin-resistant *S. aureus* (MRSA), are extremely resistant to antibiotics, making the fight against them difficult. *E. coli*, normally inhabiting the intestines, can, however, cause pathology ingested into other human body organs or cavities. *P. aeruginosa* can be stable to many classes of antimicrobial and invoke infections, especially in patients with weakened immunity [136]. Despite the antimicrobial mechanism of SeNP action not having been fully investigated, there are a number of well-established assumptions. The SeNPs’ biocidal properties depend on their size and shape: spherical nanoparticles of smaller size—50–100 nm—are more effective than larger nanoparticles because they can easily penetrate into the bacterial cell/membrane and affect the bacterial biological activity [136,155]. SeNPs are active against both Gram-positive and Gram-negative microorganisms [156]. The more negative membrane charge of the Gram-negative bacteria formed from lipopolysaccharides, as well as the cytoplasmic membrane and the outer cell membrane containing a thin peptidoglycan layer between them with a periplasmic compartment, allows selenium nanoparticles to bind effectively to bacterial cells [136,157]. At the same time, SeNPs possess high antimicrobial activity against *S. aureus* and *B. subtilis*, probably due to a thicker layer of peptidoglycans and the porin presence [36,83,139]. Besides, Tran et al. have assessed the antibacterial mechanism of SeNPs in Gram-positive and Gram-negative bacteria and concluded that SeNPs have strong electrostatic repulsion toward the lipopolysaccharide and membrane of Gram-negative bacteria, which is highly negative in nature [158]. Filipovic et al. showed that negatively charged SeNPs exhibit more pronounced activity towards Gram-positive bacteria, which may be due to the absence of negatively charged LPS molecules in their cell walls and the absence of electrostatic repulsion [159], while the negative charge presence on SeNP surfaces can cause electrostatic repulsion from the lipopolysaccharide part of the Gram-negative bacterial cell wall, making them less susceptible to the tested SeNPs than Gram-positive bacteria [132,136]. Additionally, the Se ions antimicrobial action has been suggested to depend on their absorption and accumulation into microbial cells, which drive the cytoplasm membrane shrinkage and cell bioactivity inhibition [136,160]. The SeNPs’ interaction with a bacterial cell triggers the integrity violation of the membrane, and SeNP penetration inside causes bacterial lysis. In addition, their released interior components are apparently attached to SeNPs [136,160]. The bacterial cell membrane loses its integrity due to phospholipid bilayer destruction, where nanoparticles interact with membrane proteins and inactivate them by reducing membrane permeability or even reacting with thiol or sulfhydryl groups present in membrane proteins, thus denaturing them [138,143]. For example, during interaction with nanoparticles, the surface potential of the *S. aureus* bacterial membrane was weakened and neutralized, leading to an increase in surface tension. At a high concentration of SeNPs, interactions adduce a change in surface tension, leading to membrane depolarization at the point of contact. As a result, bacterial membranes exhibit abnormal structures, such as membrane rupture, membrane swelling, torn cells often found in the form of aggregates or clots, cell shrinkage, shape irregularity, and increased roughness [76].

Apparently, more complex mechanisms are involved in the process: interaction with vital cellular components (RNA, DNA and ribosomes) in order to change and deactivate their intracellular processes; ATP depletion and a change in the membrane’s polarity; and the ROS generation and free radical formation (hydroxyl radicals, superoxide anions, and hydrogen peroxide) after penetration through bacterial membranes, which can damage DNA and proteins through oxidative stress, to enhance the peroxidation of membrane lipids and subsequently increase the cytoplasmic contents’ leakage, suppressing metabolism and leading to cell death [156]. Thus, in experiments with *P. aeruginosa*, *E. coli*, *Vibrio parahemolyticus*, *S. aureus*, *B. cereus*, and *B. subtilis* enhanced protein leakage was demonstrated, moreover, bio-SeNPs may accelerate proteins and polysaccharide leakage from bacterial cytoplasm [160]. It is supposed that the negative charges discovered on the protein content in bacterial walls might cause interaction with ionic species generated by the existence of SeNPs [161]. Martínez-Esquivias et al. obtained interesting data by in vitro and in silico analysis: *S. aureus* growth inhibition due to changes in cysteine, methionine and arachidonic acid metabolism, which are considered targets for drug development against *S. aureus*, was found [110].

Undoubtedly, capping agents are of great value in the SeNPs antibacterial activity. For example, anacardiac acids from *Amphipterygium glaucum*, inhibitors of bacterial histidine protein kinase in two-component regulatory systems, can act as capping agents. These proteins participated in the expression regulation of the virulence factor through quorum sensing (a mechanism of communication between unicellular organisms) [162]. It is assumed that the biologically active substances (BAS) from *C. officinalis* extract present on the SeNPs surface are capable of damaging the signaling receptors necessary to carry out quorum sensing [163]. The SeNPs coating of compounds such as flavonoids, terpenoids, alkaloids and other BAS from plant extracts is a possible reason for their antimicrobial potential because they can inhibit enzymes responsible for DNA replication and gene expression necessary for the microbe’s survival. In addition, these compounds can change the cell wall and/or cell membrane permeability, as well as via the ROS generation or enzyme inactivation, leading to microbial cell death [117,137].

The challenges of the fight against biofilm formation by microorganisms in medicine are very acute (for example, such as colonization on central venous catheters (CVCs), lower respiratory tract infections (due to contaminated ventilators), and catheter-related ascending urinary tract infections), because of their antimicrobial drug resistance is extremely high. It is associated with the slow or incomplete penetration (due to the polysaccharide matrix) of antibacterial drugs into the biofilm and the subpopulation emergence of dormant forms (persistors) [164].

It is believed that more than 80% of all chronic human infectious diseases are associated with biofilms [165]. In this regard, SeNPs, having antibacterial properties, can become potential agents against biofilm formation. Thus, SeNPs (with an average particle size of 28 nm) produced by *P. vermicola* have an antibiofilm effect on *S. aureus*, *B. cereus*, *E. coli* and *Salmonella enteritidis*, which lose their ability to form a biofilm 95% higher [76]. SeNPs were able to inhibit biofilm formation as well as disaggregate mature glycocalyx (glycoprotein and glycolipid cover), ultimately led to the bacterial cell death in *P. aeruginosa* [166]. The biofilm degradation of *E. coli*, *S. aureus* and *P. aeruginosa* [67], *Salmonella typhi* [167], *P. mirabilis* [168], *Enterococcus faecalis*, *Salmonella typhimurium*, and *S. enteritidis* [169], *Klebsiella pneumoniae* [139], *Acinetobacter* sp. [18] was shown. Furthermore, the most expressed activity was detected for dual-species biofilms grown from *S. aureus* and *Candida albicans*, where inhibition was greater than 90% [159].

**Figure 3:**
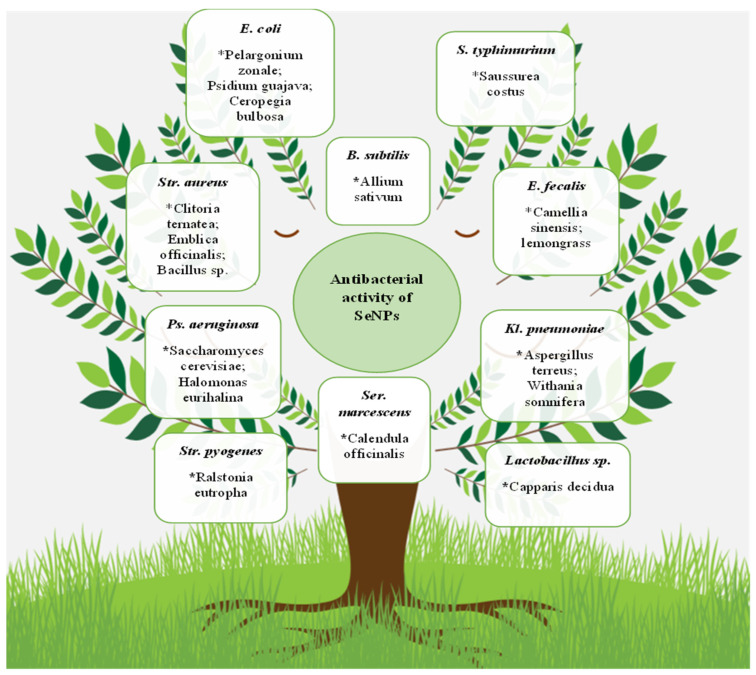
Antibacterial SeNP activity. The biosources of selenium nanoparticles are indicated by stars. * *Pelargonium zonale*; *Psidium guajava*; *Ceropegia bulbosa*; *Allium sativum*; *Saussurea costus*; *Clitoria ternatea*; *Emblica officinalis*; *Bacillus* sp.; *Camellia sinensis*; lemongrass; *Saccharomyces cerevisiae*; *Halomonas eurihalina*; *Ralstonia eutropha*; *Calendula officinalis*; *Aspergillus terreus*; *Withania somnifera*; *Capparis decidua*.

It should be noted that, in general, the antibacterial properties’ multiformity of selenium nanoparticles are huge. SeNPs overwhelming effect on microorganisms, including the Gram-positive bacteria *S. aureus* [56,170,171,172,173,174,175,176,177,178,179,180,181,182], *B. subtilis* [36,120,156,178], *Streptococcus mutans* [179,181], *E. faecalis* [106], *Mycobacterium tuberculosis* [183,184], and *Micrococcus luteus* [185], and the Gram-negative *E. coli* [120,138,172,186,187], *S. typhimurium* [95,117,155,188], *S. typhi* [189], *P. aeruginosa* [67,141,156], *Klebsiella pneumoniae* [123,190], *P. mirabilis* [110,124], *Vibrio harveyi* and *Vibrio parahaemolyticus* [191], *Shigella* sp. [101], and *Serratia marcescens* [163], was demonstrated. Some of these microorganisms and nanoparticle bioproducers are presented in Figure 3.

The minimum inhibitory concentration (MIC) is the lowest concentration that has the efficacy to inhibit microbial growth. Standard antibiotics are used as positive controls. Another way is to evaluate the inhibition zone or inhibition concentration. Data on the antibacterial properties of some selenium nanoparticles are presented in Table 2.

### 3.2. Antifungal Activity

Despite the development and introduction of new antifungal drugs, the problem of increasing microorganism resistance to them still needs to be solved. Fungi such as *Candida* yeast and filamentous fungi like *Aspergillus* are widespread and bring huge costs to health systems in different countries. They exist as opportunistic human microorganisms with a suppressed and defective immune system, and some strains have high resistance to antifungal drugs. The nanoparticle application is one of the new ways to tackle this problem. The proposed mechanism of antifungal action is similar to those of bacteria: the smaller size and high ratio of surface area to volume allow them to interact closely with the cell membranes of microorganisms (reaction with a thiol (-SH) protein group affects membrane permeability), which facilitates interaction and intracellular diffusion, causing significant membrane damage and having a toxic effect on DNA, disrupting the mitochondrial membrane, changing gene expression, or inhibiting cell proliferation by ROS [107]. For example, when SeNPs were exposed to *F. oxysporum* and *Colletotrichum gloeosporioides*, a change in the morphology of fungal colonies was observed, and the amount of mycelium and the cottony shape of the fungus were reduced [107]. The effect on the fungal cells led to the appearance of uneven bloating and mycelium fragmentation with softening of their walls and the appearance of deformation signs, and later the entire fungal mycelium was mostly lyses and lost its distinctive structure; the interior cellular components leaked outside the hyphae [107]. SeNPs from *Candida glabrata* were found to attach and agglomerate on the surface of cells, causing the loss of membrane smoothness, appearance of bulges, and impairment of the membrane’s integrity [192]. The antimycotoxin activity of selenium nanoparticles against ochratoxin A (OTA) of mycotoxigenic *Aspergillus ochraceus* and *Penicillium verrucosum* [130] was detected. *Trichoderma harzianum*-mediated SeNP treatment significantly reduced the production of fumonisin B1 and Alternaria toxins, hyphae and spores demonstrated great damage, and oxidative stress and the gene expression of FUM1, PA, TRI5, and TRI6 were substantially decreased [35].

The biofilm formation of *C. albicans* is a separate medical problem. It was found that bio-SeNPs from *Paenibacillus terreus* are able to suppress the gene expression involved in morphogenesis and biofilm formation in *C. albicans* [72]. Adhesion was the first step for the initiation of biofilm formation in *C. albicans* and was mediated by the expression of Als genes that encoded the adhesion proteins that helped the cells adhere to the hydrophobic surfaces or attach themselves to the endothelial and epithelial cells. Als3, 4, and 6 genes were downregulated in the presence of SeNPs, and the Hwp1 gene that was involved in hyphal growth and biofilm formation was also downregulated. The suppression of the Efg1 gene probably led to the suppression of the hyphae cell wall gene expression, such as Hwp1 and Als3. It was observed that the expression of the Phr1 gene inhibited the transition to hyphae, and suppression of the Phr1 gene regulation probably inhibits morphogenesis in *C. albicans* in the presence of selenium nanoparticles [72]. In addition, SeNPs probably reduce the ergosterol content, an important component of the plasma membrane’s uniformity, rigidity, fluidity, and integrity, and thereby cause fungus death due to membrane damage [130]. The homeostasis and biosynthesis of ergosterol are crucial for cellular processes such as the transcription regulation of biosynthetic pathway genes and proteins involved in sterol processing and absorption, which are of fundamental importance for fungi growth. The ERG11 gene, the target of azole antifungals, catalyzes an intermediate step in the biosynthetic manufacture of ergosterol in fungi. The enzyme sterol Δ5, 6-desaturase is encoded by the ERG3 gene, and the mutation of ERG3 has been associated with increased cross-resistance to polyene antifungal agents in yeast species. Polyene antifungals bind to ergosterol in the plasma membrane and form large pores that impact cell function [193]. SeNPs were found to reduce the expression of resistance genes to the antifungal drugs ERG3, ERG11, and FKS1 in *C. albicans* and *C. glabrata*, associated with the virulence of opportunistic yeast [193].

Capping agents are of great interest, in particular those produced by plant phenolic substances such as gallic acid, thymol, tannins, alkaloids, flavonoids (particularly catechin), polyphenols such as tannins, terpenoids, and saponins, already famous for their fungicidal properties [194]. The antifungal activity of selenium nanoparticles is also shown in relation to *Aspergillus fumigatus*, *Aspergillus niger*, *Aspergillus flavus* [195], *F. oxysporum*, and *P. chrysogenum* [196] (Table 3).

### 3.3. Antiviral

Viruses are non-cellular infectious agents that pose one of the greatest threats to humanity, and the COVID-19 pandemic became direct proof of that. Newly available antiviral drugs with a minimal toxic effect, especially broad-acting, could be the key to solving a lot of medical problems. Unfortunately, there is not much information about biosynthesized SeNPs yet, but they also inspire serious hope in the struggle against various human viruses. Dengue fever, a viral infection transmitted by humans through infected mosquito bites, became a global problem after World War II and spread to more than 110 countries, mainly in Asia and South America. Thus, it was shown that selenium nanoparticles synthesized by the actinobacterium *Streptomyces minutiscleroticus* had antiviral activity against type 1 dengue virus [170]. The antiviral activity tended to increase with an increase in dose, and concurrently a reduction in viral growth was shown [170]. It is especially necessary to note the antiviral effect of *Portulaca oleracea*-based SeNPs on the inhibition of the hepatitis A virus (HAV) and Coxsackie B virus (Cox-B4), which are widespread everywhere and can trigger severe complications [114]. Al-Haggar et al. discovered antiviral activities against adenovirus-9 for *Bacillus niabensis*-mediated SeNPs [197]. Selenium nanoparticles synthesized by the brown algae *Polycladia myrica* have good antiviral activity against the hepatitis A virus [99].

**Figure 4:**
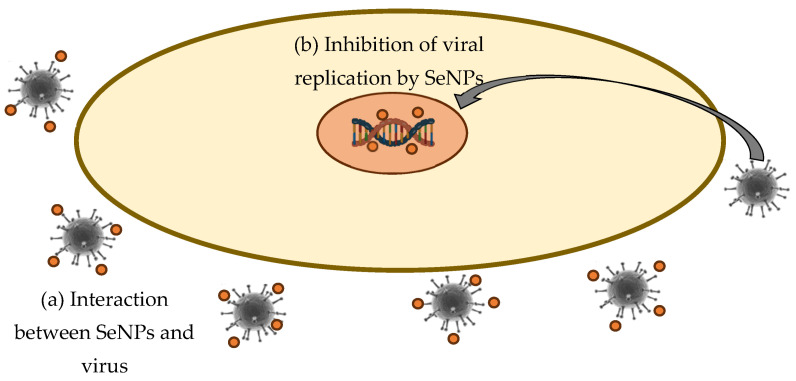
Proposed mechanism of SeNPs’ antiviral activity.

The exact antiviral mechanism of selenium nanoparticles is still to be explored; however, based on data on chemically produced SeNPs (most often with vitamin C participation), it is assumed that antiviral activity is associated not only with direct virus destruction but also with its role in regulating selenoprotein function, making SeNPs ideal candidates for antiviral drugs with a wide spectrum of antiviral activity [198]. Apparently, nanoparticles are able to penetrate directly into the host cell, besides direct interaction with the virus surface glycoproteins, and exhibit their antiviral activity by binding to viral factors and host cellular factors, thereby blocking the virus replication mechanism [199]. SeNPs probably resist the proliferation of viruses in Vero cells and inhibit the apoptotic protein activation by viruses inside host cells [114]. It was shown that SeNPs can effectively inhibit the production of ROS by the H1N1 virus [200]. H1N1 induced intracellular apoptosis in MDCK (Madin Darby Canine Kidney) cells, and SeNPs inhibited the generation of caspase-3-mediated apoptosis by increasing the Gpx1 (glutathione peroxidase 1) level by regulating the apoptosis-signaling pathway [200]. SeNPs could prevent H1N1 from infecting MDCK cells and causing cell apoptosis by blocking chromatin condensation and DNA fragmentation, along with the inhibition of ROS generation and activation of p53 phosphorylation [201]. It was found that SeNPs loaded with oseltamivir prevented the host cell apoptosis induced by EV71 (Enterovirus 71) infection through the mitochondrial pathway and reduced the generation of reactive oxygen species [202]. Also, SeNPs could inhibit H1N1 influenza virus-induced apoptosis by inhibiting ROS mediated Akt (Protein kinase B) and p53-signaling pathways [203]. In addition, Se was reported as a modulator of specific enzymes responsible for some key biological interactions and ROS elimination, and selenium deficiency can increase susceptibility to viral infection [204]. In this regard, selenium nanoparticles could replenish the selenium reserve in the body while simultaneously exerting an antiviral effect [204]. This opens up ample opportunities for testing SeNPs in vivo. The proposed mechanism of SeNPs’ antiviral activity is presented in Figure 4.

The SeNPs’ antiviral properties can be extremely useful, especially in the confrontation of pandemic viruses like COVID-19. Selenium can play an important role here as a trace element necessary for normal life. Firstly, Se deficiency leads to a decrease in selenoprotein expression regulation, which, in turn, results in the immune system weakening against infectious diseases. During viral infections, the host’s metabolism can be disrupted in several ways, leading to a regulation violation of redox homeostasis. On the other hand, a severe inflammatory reaction and an uncontrolled immune response can cause a massive cytokine storm, threatening the COVID-19 patients’ lives.

### 3.4. Anticancer

Cancer has become a real scourge of humanity over the past hundred years. The progressive number of cancer cases all over the world is of serious concern and requires a speedy way out of this situation. The new strategies for cancer treatment imply improving drug targeting as well as reducing their toxicity. The nanotechnological approach seems to be one of the most promising in this context. Successful results were shown in SeNP studies to induce cytotoxicity in cancer cells. Although the molecular mechanism of the SeNPs’ antitumor effect has not been fully revealed, research is in an active phase.

Among the SeNPs’ mechanisms of antitumor action in vitro, the greatest attention is paid to apoptosis, characterized by nuclear chromatin condensation, cytoplasm compression, and membrane dysfunction. It was found that oxidative stress and ROS generation are the main cytotoxicity mechanisms induced by SeNPs, where ROS modulates apoptosis by regulating the enzymatic activity involved in cell death pathways. The cancer cells exhibit an acidic pH and an imbalanced redox state. These conditions in cancer cells initiate the pro-oxidant conversion of SeNPs and trigger the development of free radicals in malignant cells. This leads, on the one hand, to mitochondrial membrane destruction, causing mitochondrial protein leakage, and, on the other hand, to stress in the endoplasmic reticulum (ER). The mitochondrial membrane destruction results in the outflow of various proteins and triggers apoptosis through caspase activation (a family of protease enzymes playing an important role in programmed cell death) [205]. Selenium nanoparticles can stimulate p53 expression in cancer cells, leading to caspase-9 activation, mitochondrial membrane potential depletion, and the induction of apoptosis. In addition, in cellular processes, DNA structure is damaged, causing the cell cycle to stop and, ultimately, cell death. Additionally, selenium nanoparticles can penetrate cancer cells through endocytosis. It is assumed that SeNPs in endocytic vesicles can penetrate into mitochondria through the mitochondrial membrane fusion pathway, mediated by the TLR4/TNF protein complex associated with the factor 3 (TRAF3)/mitofuzin-1 (MFN1) receptor [206]. The secretion of a large amount of lactate anions from cancer cells and the exposure of more phospholipids on the surface of breast cancer cells lead to a negatively charged surface of cancer cells as compared to fibroblasts; thus, positively charged SeNPs may have a strong affinity for breast cancer cells, causing the enhanced anticancer efficacy of SeNPs [41].

ROS are highly reactive molecules, including oxygen ions, free radicals, and peroxides. ROS are produced as a natural byproduct of normal cellular metabolism and play a significant role in cell signaling. ROS can chemically bind to nucleic acids and proteins; therefore, ROS generation is a substantial cellular event induced by Se compounds and results in cell apoptosis and/or cell cycle arrest [207]. It was found that SeNPs fabricated in *Undaria pinnatifida* polysaccharide caused a dose-dependent increase in the intracellular level of ROS, the most important mediator in the induction of apoptosis in vitro for A375 human melanoma cells, as well as DNA fragmentation, an important biochemical sign of cellular apoptosis [208].

The mitochondrial respiratory chain is a potential source of ROS, such as superoxide and hydrogen peroxide. The loss of mitochondrial membrane potential is associated with caspase activation and the initiation of the apoptotic cascade. When mitochondrial membrane integrity is violated, cytochrome C moves from the mitochondria to the cytosol, triggering a signaling caspase cascade that damages DNA. Additionally, ROS control the translocation, phosphorylation and cleavage of the pro-apoptotic protein Bcl-2, which induces apoptosis. Bcl-2 is one of the key apoptosis proteins involved in the mitochondrial pathway, which exerts its bioactivity by inhibiting the release of cytochrome C. The decrease in the Bcl-2 expression level results in increased mitochondrial membrane permeability and the release of apoptosis activators such as cytochrome C. The increased cytochrome C quickly induces the activation of caspase-9 [205]. For example, an increase in membrane potential depletion due to the use of SeNPs induced apoptosis in A375 cells through mitochondria-mediated pathways [208]. The caspase enzyme is mainly realized through the mitochondrial/Cyt c pathway, the death receptor pathway, and the endoplasmic reticulum pathway.

The main role of caspase-9 is in the internal transmission of the apoptosis signal, while caspase-8 is involved in the external apoptosis signal transmission, and caspase-3 is a decisive factor in apoptosis implementation. Thus, an increase in the activity of caspase-3, the most important signaling module of the apoptosis cascade that plays a vital role in protein breakdown, provokes the programmed destruction of lung cancer cells, as was demonstrated for *E. coli*-mediated SeNPs [209]. Treatment with Carvacrol-mediated SeNPs directly targeted Bcl-2, Bax, and caspase-3, leading to the mitochondrial leakage of cytochrome C and subsequent activation of the apoptotic cascade in MCF-7 cells [151]. SeNPs dose-dependently increased caspase-3 levels in cancer cells; moreover, in normal cells, the SeNP concentration necessary to induce an increase in the caspase-3 level was found to be significantly higher compared to RAW 264.7, Caco-2, MCF-7, and IMR-32 cancer cells [130]. SeNPs from *Lactobacillus casei* treatment increased the expression of caspase-3, p53, and Bax mRNA and decreased the mRNA expression of bcl-2 in HepG2 cells [78]. The activation of both caspase-3 and caspase-9 suggested that ferulic acid (from *Ferula foetida*-modified SeNPs) induced the apoptosis of HepG-2 cells via the intrinsic mitochondrial pathway; moreover, they are able to interact directly with DNA and inhibit replication and transcription in HepG2 cells [210]. Increased caspase-3 activation was detected, and two different receptors initiating apoptosis, TRAIL-R2 and Fas, were increased after SeNPs impact [211]. Also, transcription factors p53 and p27 were both enhanced in treated CT26 cells (mouse colon carcinoma cells). Additionally, nanoparticles induced the modulation of the expression of various proteins associated with apoptosis [211]. The molecular mechanism of *Sargassum wightii* SeNP-induced A549 cell apoptosis was found to be through overproduced ROS-mediated activation of p53- and AKT-signaling pathways [212]. SeNPs were effective for activating various mitochondrial pathways that enhance apoptosis and inhibit the autophagy mechanism. In addition, laminarin polysaccharide-decorated SeNPs reduced or stopped Bcl-2 expression (antiapoptotic factor) and reduced Bcl-2’s negative effect on Beclin-1 (autophagy cell system protein) in HepG2 cell lines [213]. SeNPs induced by hawthorn fruit increased caspase-9 regulation and Bcl-2 downregulation [214].

Recently, additional evidence was found for the novel role of microRNAs in the cell cycle and cancer cell apoptosis. Most microRNA functions are implemented by suppressing their target genes. miR-16 has been reported to be closely related to cell cycle regulation and apoptosis. *E. coli*-mediated SeNPs enhance the regulation of miR-16 by the regulation of the reduction in miR-16 in two key targets: cyclin D1 and Bcl-2. It may be a direct pathway for SeNPs to promote prostate cancer cell apoptosis [215].

It was also shown that SeNP cell treatment contributed to the release of HMGB1 (a protein from the group of nuclear non-histone proteins interacting with cell DNA) from the nucleus and its subsequent secretion by dying cells, as evidenced by an increased level of this protein in the cancer cell cytoplasm and a corresponding decrease in the nucleus [211].

The significant induction of oxidative stress markers, such as ROS, 8-OHDG, LPO, and NO, was accompanied by a decrease in antioxidant marker levels (CAT, SOD, GPx activity and GSH levels) in MCF-7 cells exposed to green SeNPs, in contrast to control cells [151]. Possessing antioxidant properties, GSH not only protects the cell from toxic free radicals, but also generally determines the redox characteristics of the intracellular environment. It was found that ROS generation converts GSH to GSSG (glutathione disulfide) through the oxidation process. Oxidized glutathione is reduced by the enzyme glutathione reductase induced by oxidative stress. The ratio of reduced and oxidized glutathione formed in the cell is one of the most important parameters showing the oxidative stress level [207]. SeNPs increased oxidative stress and reduced the level of endogenous antioxidants such as GSH, superoxide dismutase, catalase and GSH peroxidase in MCF-7 cells [211]. *Spermacoce hispida* SeNP treatment decreased the levels of endogenous antioxidant such as GSH, superoxide dismutase, catalase, and GSH peroxidase [213] in the hepatocarcinoma cell line (HepG2), and also induced cell cycle arrest at the sub-G1 phase and subsequently led to apoptosis [216]. SeNPs from *Carica papaya* effectively influenced the viability of cancer cells through lactate dehydrogenase (LDH) leakage, meaning damage to the cell membrane. In addition, SeNPs selectively affect LDH leakage and membrane disruption in cancer cells because the SeNP concentration required to influence LDH leakage in normal cells is much higher compared to that in cancer cells [130].

The proliferating cell nuclear antigen (PCNA) is called the “ringmaster of the genome” because it regulates the cell cycle and participates in DNA synthesis, suggesting its active role in oncogenesis. Therefore, PCNA is widely used as a cell proliferation marker in both healthy and malignant tissues since it usually reflects cell proliferation activity [151]. Interestingly, SeNPs reduced the PCNA expression level in MCF-7 cells, showing their role in suppressing oncogenesis and proliferation in breast cancer by inhibiting PCNA gene expression [151].

Cancer cells (MCF-7) treated with *Diospyros montana*-mediated SeNPs demonstrated chromosomal instability and mitosis delay. SeNPs also reduced their cell viability, leading to the loss of intercellular contact, cell shrinkage, and apoptotic body formation [121]. SeNPs from *Acinetobacter* sp. caused a dose-dependent cell number decline in 4T1 cells (breast cancer cells), as well as a loss of intercellular contact and cell shrinkage [41].

Notably, SeNPs synthesized by *Bacillus licheniformis* induced necroptosis in PC-3 cells [217]. These nanoparticles evoked mitochondrial damage without affecting cell membrane integrity. Sonkusre and Cameotra observed SeNP endocytosis, triggering a dramatic mitochondrial ROS production that, along with ATP depletion, indicated SeNP-induced mitochondrial damage [214]. Moreover, an increase in the expression levels of TNF and IRF1 genes, involved in the regulation of necrosis (necroptosis) induction, was found. Additionally, SeNPs induced cell death in PC-3 cells by the ROS-mediated activation of necroptosis, independent of RIP3 and MLKL and regulated by an RIP1 kinase [217].

Autophagy is a biological process whereby the internal components of the cell are transported to their lysosomes or vacuoles and are subsequently degraded there. SeNPs using *Kaempferia parviflora* (black ginger) also caused the autophagy of AGS by increasing the autophagic flux marker protein, LC3B-II, while inhibiting the autophagic cargo protein, p62 [218].

Capping agents, having their own antitumor abilities, can be of great importance in green SeNPs’ anticancer activity. Nanoparticle capping can enhance their absorption via accumulation by endocytosis in cancer cells, which can therefore lead to ROS generation induction, the disruption of the mitochondrial membrane potential, and mitochondrial-mediated apoptotic pathway stimulation through caspase activation and cell growth arrest in the G2/M phase [132]. It is assumed that tetramethoxyflavone, dimethoxyflavone, and trimethoxyflavone from black ginger ethanol extract as capping agents have anticarcinogenic properties and can thus contribute to the antitumor activity of SeNPs [218].

**Figure 5:**
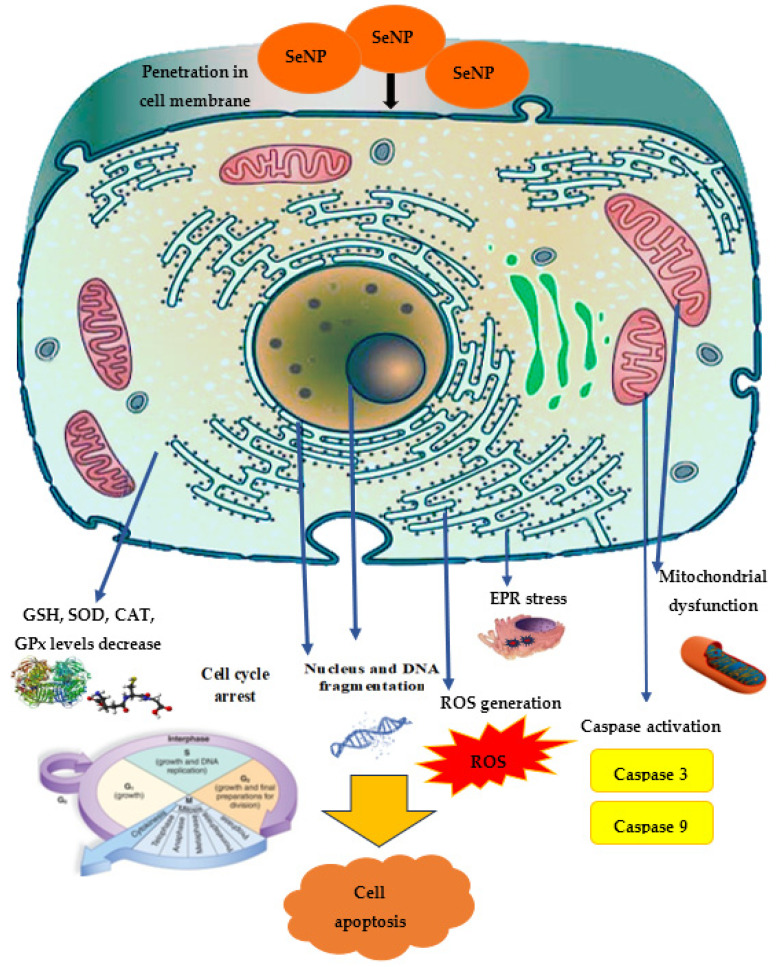
The proposed mechanism of SeNPs’ anticancer activity.

In general, the spectrum of SeNPs’ anticancer activity demonstrated in vitro is quite wide: in leukemia cells [219], MCF-7 [124,220], AGS (human gastric adenocarcinoma cell line) [111], HeLa [64,170], HepG2 [138,221], 4T1 [222], SW480 (human colorectal adenocarcinoma cell lines) [223], MDA-MB-231 (mammary gland adenocarcinoma cell lines) [224], and A2780 cells (human ovarian cancer cell line) [225]. The proposed mechanism of anticancer effect is demonstrated in Figure 5. 

In vivo experiments demonstrate that SeNPs against Ehrlich ascites carcinoma cells cause a significant decrease in tumor volume and massive tumor cell necrosis, improve kidneys and liver functional parameters (urea and creatinine, as well as liver enzymes ALT and AST), and reduce cancer cell metastasis and apoptosis [206].

A decrease in SOD and glutathione (GSH) activity in mice livers carrying tumors was also shown, which was an indicator of mitochondrial loss and the inhibitory activity of superoxide dismutase, manifested as a result of a decrease in tumor growth [206]. The SeNPs’ inhibitory effect was determined on tumor growth; the upregulation of LC3II and caspase-3 proteins was also found in the tumor tissues of 5-FU-treated mice; no pathological phenomena occurred, such as cell edema and organelle disintegration, and there was no obvious histological liver or kidney damage [218].

### 3.5. Antioxidant Activity

Hydrophilic/lipophilic, enzymatic/non-enzymatic compounds act as antioxidants, inhibiting oxidation by neutralizing the oxidative action of free radicals and other substances. Antioxidant activity was found for many nanoparticles, including SeNPs [226]. Green SeNPs’ ability to neutralize these free radicals can be explained by the nanoparticle dispersibility in the medium owing to their tiny particle size, in addition to high chemical activity, as well as the antioxidant activity of the covering biomolecules. The antioxidant capacity analysis in vitro is usually realized with the help of such molecules as 1,1-diphenyl-2-picryl-hydrazyl (DPPH), where the free electron of nitrogen in DPPH is reduced by hydrogen present in antioxidants, ABTS (2,2′-azinobis (3-ethylbenzothiazoline-6-sulfonic acid)) for checking both hydrophilic and lipophilic antioxidants, and the FRAP assay. The ferric reduction antioxidant power (FRAP) assay is based on electron transfer rather than hydrogen transfer, unlike DPPH and ABTS [227]. For example, SeNPs from *Withania somnifera* and *Emblica officinalis* demonstrated powerful dose-dependent activity to remove DPPH and ABTS radicals [123,129]. Presumably, the high antioxidant properties of SeNPs may be due to selenium because it plays a main role in increasing selenium enzyme activity, such as glutathione peroxidase, and helps protect cells and tissues from free radicals [129]. Selenium nanoparticles from Aloe vera showed reducing activity against FRAP [119]. Antioxidant potential was shown for the endophytic fungi SeNPs: DPPH could be effectively scavenged with antioxidants using the donation of hydrogen to form a stable DPPH molecule [91,228]. SeNPs were significantly active toward ABTS, which is applicable to both hydrophilic and lipophilic antioxidant systems [229,230].

An excess of ROS can provoke irreversible oxidative damage to proteins, DNA, and cellular lipids in the human body. ROS, most often represented by superoxide anionic radicals—(O_2_^−^), hydroxyl radicals (·OH), and hydrogen peroxide (H_2_O_2_), can play a role in the initiation of diseases such as diabetes mellitus, Alzheimer’s disease, coronary heart disease, cancer, arteriosclerosis, and other health disorders associated with aging. Antioxidants protect the human body from oxidative damage and are able to slow down the progression of these processes to a certain degree by reacting directly with free radicals or improving antioxidant enzyme activity. Literature data indicate that SeNP treatment significantly inhibited intracellular ROS production in IPEC-J2 cells exposed to H_2_O_2_ and reduced the MMP loss (mitochondrial membrane potential) caused by oxidative stress induced by H_2_O_2_ [231]. Such an effective mitigation of the H_2_O_2_-induced oxidative damage effects of IPEC-J2 may be associated with an increase in the antioxidant enzyme levels, including GPx and SOD [231]. Selenium nanoparticles from *B. paralicheniformis* can protect IPEC-J2 cells from oxidative stress by inhibiting ROS production [79]. SeNPs from *Lycium barbarum*, having positive antioxidant activity, protected PC12 from toxicity induced by H_2_O_2_ [226]. The antioxidant activity of selenium nanoparticles from *Pantoea agglomerans* was demonstrated using primary cultures of umbilical vein endothelial cells (HUVECs) [61]. SeNPs from tea extracts showed a higher ability to scavenge hydroxyl radicals [109].

The in vivo studies confirmed the data in vitro. For example, SeNPs from Moringa peregrina significantly restored the SOD (superoxide dismutase) activity in blood serum and liver homogenates and also increased the specific activity of CAT (catalase) in lipopolysaccharide (LPS)-induced oxidative stress mice. Moreover, they caused a decrease in the carbonyl content in blood sera, and were able to eliminate damage caused by oxidation in the body and protect its organs from necrosis and dysfunction [232]. Interesting data were obtained in the work of Qu et al., who found that SeNPs synthesized by *B. licheniformis* counteract the toxicity of deoxynivalenol (DON). DON is vomitoxin, one of several mycotoxins produced by certain fusarium types that often affect corn, wheat, and other crops in the field. DON could increase the production of free radicals, thus initiating oxidative stress. Green SeNPs improved GPx and T-AOC (total antioxidant capacity) levels in laying hens, increased egg production, significantly decreased the number of eggs with soft shells or cracks, reduced the DON effect on blood parameters, increased the calcium content in blood serum, and generally diminished oxidative stress in the chicken body [233]. Selenium nanoparticles from *Rhodopseudomonas palustris* were capable of defending mice from CCl4-induced liver damage by increasing the antioxidant ability to suppress oxidative stress [234]. Hepatoprotective effects may be associated with the antioxidant defense system level, including enzymatic antioxidants such as SOD, CAT, and GSH-Px, which are significantly increased with SeNP injection. In addition, the malondialdehyde (MDA) levels in the liver of mice treated with CCl4 significantly decreased [234].

Capping agents may be of vital importance in antioxidant activity implementation. For example, the IPEC-J2 cell protection from oxidative damage caused by H_2_O_2_ can be explained by the increased antioxidant activity of SeNPs due to exopolysaccharide surface coating [79]. The SeNPs’ high antioxidant activity may be associated with the biomolecules’ involvement in the cell-free *Geobacillus* sp. extract [235].

### 3.6. Antidiabetic Activity

Diabetes mellitus is a group of endocrine diseases conjugated with impaired glucose uptake owing to absolute or relative (violation of interaction with target cells) insufficiency of the hormone insulin. As a result, hyperglycemia develops—a persistent increase in blood glucose. The increase in disease cases on the planet causes serious concerns in medicine practice and requires the search for new, high-quality drugs with minimal side effects. The malady is associated with oxidative stress, reactive nitrogen species production (RNS) and ROS generation, accompanied by inflammation, β-cell dysfunction, hyperlipidemia, insulin resistance, and impaired glucose tolerance, leading to diabetic complications. Therefore, biocompounds with antioxidant activity seem to be the most suitable way to solve this problem. Literature data indicate that SeNPs produced by biochemical methods (most often by the reduction of sodium selenite with glutathione in the presence of bovine serum albumin (BSA)) raise the insulin concentration in the blood serum in mice with diabetes mellitus [236]. SeNPs could significantly decrease hepatic (serum ALT, AST, and ALP) and renal (serum uric acid, urea, and creatinine) function markers, total lipid, total cholesterol, triglyceride and low-density lipoprotein cholesterol levels, and glucose-6-phosphatase activity. At the same time, SeNPs increased malic enzyme, hexokinase, and glucose-6-phosphate dehydrogenase activity, liver and kidney glycogen contents, and high-density lipoprotein cholesterol levels. In addition, SeNPs were able to prevent histological injury in the hepatic and renal tissues of rats [236]. Presumably, the risk of diabetic complications can be minimized in this way, and this selenium nanoparticle effect is related to the free radical’s absorption and/or their insulinomimetic effect [236]. The SeNPs’ antioxidant activity has the ability to restrain the progression of diabetic nephropathy, a common leading cause of end-stage renal failure (ESRD) in type 1 diabetes mellitus [237]. In [237], male Sprague Dawley (SD) rats injecting streptozotocin (STZ) were treated with SeNPs. SeNPs effectively reduced blood levels of urea nitrogen, creatinine, fibronectin, and collagen, and increased albumin levels in diabetic rats. Furthermore, treatment with selenium nanoparticles had an effect on oxidative stress parameters: it significantly reduced MDA levels and prevented a decrease in GSH in animal kidneys [237]. SeNPs elevated the levels of heat shock protein HSP-70, preventing cell death under the conditions of oxidative stress caused by glucose); longevity protein SIRT1 (expression of cell cycle arrest protein p21, which negatively correlates with SIRT1 levels) was also reduced; and apoptotic proteins Bax and Bcl-2 in diabetic kidney was modulated [237]. SeNPs also contributed to the strengthening of the elimination of damage induced by oxidative stress caused by diabetes by reducing LPO and NO levels in the pancreas. In addition, the antioxidant enzyme activity of GPx and GSH levels in diabetic rats were increased [238].

As for green SeNPs, very promising results were discovered, too. In in vitro experiments, selenium nanoparticles produced using *Acacia catechu* had an inhibitory effect on alpha-amylase, regulating blood glucose levels [239]. SeNPs functionalized with a novel polysaccharide (RTFP-3) extracted from *Rosa roxburghii* fruit in INS-1 cells effectively blocked intracellular ROS overproduction, mitochondrial damage, the activation of caspases-3, -8, and -9 in INS-1 cells, indicating that SeNPs functioned by reducing oxidative stress and down-regulating the uncoupling protein-2 expression (UCP-2 is an uncoupling protein, functioning to disperse the proton gradient generated through the inner membrane of mitochondria, and is thus associated with a decrease in the membrane potential of mitochondria) [240]. SeNPs from *Hibiscus sabdariffa* were able to enhance the decrease in testosterone levels in the blood serum in streptozotocin (STZ)-induced diabetes rats. SeNPs can significantly reduce oxidative stress indicators in testicular tissues, such as nitric oxide and MDA, restore impaired activity of antioxidant enzymes (CAT, GR, SOD and GPx), increase glutathione activity in testicular tissues, and are also able to prevent histological damage in rat testicles [241]. *Catathelasma ventricosum*-mediated SeNPs significantly improved body weight, blood sugar, antioxidant enzyme activity, and lipid levels in STZ-induced diabetic mice [242]. Treatment with selenium nanoparticles encapsulated by flavonoids (luteolin and diosmin) restored blood glucose levels, lipid profiles, and glycogen, glycosylated hemoglobin, and insulin levels in STZ-induced diabetic mice [240]. Moreover, SeNPs showed good antioxidant activity as examined by catalase (CAT), superoxide dismutase (SOD), and glutathione peroxidase (GPx) in the liver and kidney, and can prevent damage in the liver as evaluated by aspartate aminotransferase (AST), alanine aminotransferase (ALT), and alkaline phosphatase (ALP) activities [243]. Apparently, SeNPs significantly improved the impaired glucose tolerance due to enhanced insulin resistance, as well as increased peripheral glucose utilization. SeNPs can have a hypoglycemic effect by virtue of their insulin-like effect improving glycogenesis and enhancing cellular glucose uptake [240]. It is very likely that flavonoids themselves play an important role in antidiabetic activity, exerting a synergetic effect with SeNPs. Thus, selenium nanoparticles encapsulated with the flavonoids confirm this assumption. Having their own antidiabetic properties (increased glucose uptake, reduced lipid accumulation, and lowered triglyceride, total cholesterol, and glucose levels in the blood, weakened oxidative stress, hyperglycemia, and increased serum insulin levels), they enhanced the selenium nanoparticle effect. In the liver, Naringenin/Baicalin/SeNPs increase the superoxide dismutase, catalase, and glutathione peroxidase activities, and reduce lipids and transaminases, aspartate aminotransferase, alanine aminotransferase, and alkaline phosphatase peroxidation. They improve liver function by increasing the glycogen content in the liver and muscles and protect β-cells of the pancreas from damage caused by high glucose content [153]. Playing the role of capping agents, various plant phenolic compounds can contribute to the antioxidant and antidiabetic activity of selenium nanoparticles. For example, in danio fish suffering from diabetes, treatment with SeNPs covered with a combination of medicinal plants known for their antidiabetic properties, including *Cinnamomum verum*, *Origanum majorana*, and *Origanum vulgare*, demonstrated antilipid and hypoglycemic effects, high survival, and a decrease in glucose, ROS, and lipids in the blood. Such a protective effect of SeNPs can be explained by an increase in SOD, CAT and GPx activity in the liver, providing protection of cellular components from oxidative damage [244].

### 3.7. Anti-Inflammatory Activity

Inflammation is one of the most significant initial stages in the development of many pathological conditions. In the light of ongoing research, selenium nanoparticles appear to be a possible anti-inflammatory agent. Cytokines such as IL-6, IL-8, TNF-α. and IFN-γ can act together to initiate and regulate the inflammation process. IL-6, as an essential cytokine, is involved in various pathologies that can be improved by IL-6 inhibition. The expression of cytokines is regulated by various mechanisms. The activation of toll-like receptors (TLRs) induces pro-inflammatory cytokines such as IL-6, TNF-α, and IL-1β via nuclear factor-kappa B (NF-κB). In in vitro experiments, SeNPs biosynthesized by L. lactis capped with polysaccharides significantly attenuated the increase in IL-6, IL-8, IFN-γ, and TNF-α in IPEC-J2 cells [231]. The potential application of SeNPs using *Syzygium aromaticum* against epileptic seizures and damage to the cerebral cortex was shown in pentylenetetrazol-induced epilepsy in rats [245]. Green SeNPs reduced the levels of pro-inflammatory cytokines and suppressed the activity of the glial fibrillar acidic protein, showing their inhibitory effect on epilepsy-associated inflammation, and leading to their anti-inflammatory activity against PTZ-induced neuroinflammation. The authors attribute this protective effect not only to the SeNP activity itself but also to the plant extract flavonoids, phenols, and GSH components as capping agents that can prevent free radical accumulation and reduce oxidative stress, subsequent inflammation, and apoptosis [245]. The anti-inflammatory effect of selenium nanoparticles may have the potential to reduce acute colitis symptoms. Thus, the levels of TNF-α and IL-6 in the colon in mice with DSS (dextran sulfate sodium)-induced colitis were markedly reduced by SeNPs decorated with *Ulva lactuca* polysaccharides [246]. SeNPs inhibited the activation of macrophages by suppressing the nuclear translocation of NF-κB, which drives the transcription of these pro-inflammatory cytokines [246].

The SeNPs’ anti-inflammatory effect in the area of edema can be explained by increased permeability and retention effects. Subcutaneous SeNP administration can result in the local accumulation of SeNPs in the affected area and eventually to a weakening of inflammation. Most probably, the significant number of phenols and flavonoids on the surface of SeNPs effective in inflammatory disease treatment may enhance their anti-inflammatory effect [103]. The safety mechanism of selenium nanoparticles from *Proteus mirabilis* in spinal cord injury was determined: the therapeutic dose of SeNPs remarkably protected the spinal cord integrity, improving the motor function of the hind limbs after spinal cord injury, reduced the expression of several inflammatory factors, such as tumor necrosis factor-α and interleukin-6 in vivo, and increased the production of M2-type macrophages and regulated their polarization, indicating the suppression of the inflammatory reaction [247]. Anti-inflammatory properties in vitro for green selenium nanoparticles from *Anoectochilus burmannicus* [230], *Pterocarpus santalinus* [248], *Thymus vulgaris* [249], *Maranta arundinacea* [250], *Clitoria Ternatea* [251], etc. were found. In most studies, plant extracts with long-known anti-inflammatory and generally therapeutic properties in traditional medicine were used for the SeNP synthesis.

### 3.8. Antiparasitic Activity

Parasitic diseases are a group of illnesses invoked by helminth and protozoa parasitization in various organs and systems of the human body, characterized by prolonged intoxication, toxicity, and allergic and metabolic-dystrophic disorders. They represent a very serious problem on a global scale in light of the expansion of economic ties between countries and population mobility. New eco-friendly nanotechnology methods can be used to overcome drug resistance and various modern medicine side effects for the treatment of diseases such as leishmaniasis, malaria, toxoplasmosis, and others.

Mosquitoes are vectors of many dangerous human diseases, including malaria, filariasis, West Nile and Zika viruses, dengue, Japanese encephalitis, chikungunya, and many others. *Aedes aegypti* is the main dengue fever and chikungunya vector, a deadly viral disease that affects more than 50 million people annually. This mosquito kind is widespread in tropical and subtropical zones. *Culex quinquefasciatus* is an important vector of some parasitic and arbovirus diseases such as microfilariae—pathogenic worms—and lymphatic filariasis (elephantiasis). It was revealed that green selenium nanoparticles are capable of exhibiting larvicidal activity [252]. SeNP exposure caused damage to the cells and tissues of *A. aegypti* and *C. quinquefasciatus* larvae. Such toxicity of selenium nanoparticles synthesized using *Dillenia indica* leaf extract to the larvae and pupae of two mosquito species may be due to intracellular toxic nanoparticle effects inside the cuticle and other peripheral cells. The clear penetration and accumulation of *Cupressus sempervirens*-mediated SeNPs in the exoskeleton and several disorders in *Culex pipiens* epithelial cells were shown [252]. Under *Nilgirianthus ciliatus* SeNP influence, the epithelial layer of *A. aegypti* larvae was damaged and the peritrophic membrane fragmented [253]. Hashem et al. suggest that the toxicity of SeNPs from prickly pear peel waste to mosquitoes may be connected with the small nanoparticle size, allowing them to penetrate the insect cuticle [135]. The toxicity of selenium nanoparticles can also be caused by organelles and enzyme destruction, which reduced membrane permeability, further affecting ATP synthesis, and ultimately, blocked cellular function, leading to cell death [254]. SeNP activity against instar larvae *Aedes albopictus* was in a concentration-dependent manner [92]. Intensive condition deterioration of the posterior intestine, epithelial cells, midgut, and cerebral cortex of larvae of *A. albopictus* was detected due to the penetration of SeNPs from *Ceropegia bulbosa* through the cell membrane and further reaction with membrane proteins, preventing its functioning [120]. The cellular components of the SeNP-treated larval instar, such as the nucleus, lumen, and gut epithelial cells, were affected due to the interaction between the cellular molecules, leading to damage of the cellular components [120]. The death of *A. aegypti* larvae can be induced by tissue damage as well as through the deposition of *Murraya koenigii* SeNPs in the siphon region, which leads to suffocation [255]. Larvicidal activity may also be associated with phytocompounds performing the role of capping agents in the plant synthesis of SeNPs by *Clausena dentata* [142]. The insecticidal activity of phytoplankton chemicals such as alkaloids, steroids, terpenoids, essential oils, and phenols is known. Additionally, the SeNPs’ larvicidal activity may be associated with the denaturation of sulfur-containing proteins or phosphorus-containing compounds, such as DNA, leading to organelle and enzyme denaturation, thus diminishing the cell membrane permeability and ATP synthesis, ultimately causing cellular function loss and cell death [114,142]. Also, the SeNPs’ presence inside the cell can increase oxidative stress, resulting in cell death and generating toxic ROS [89]. It is important to note that nanoparticles conjugated with plant extracts are highly toxic to mosquito larvae; however, they do not show any toxicity to non-target species, and they are safe and do not pollute the environment; therefore, they are very promising for antiparasitic use.

Leishmaniasis is a group of natural parasitic, focal, mainly zoonotic, vector-borne diseases common in tropical and subtropical countries. It is caused by parasitic protozoa of the genus Leishmania, transmitted to humans through mosquito bites. High toxicity of selenium nanoparticles was found for both promastigote and amastigote forms of *L. major* [256]. Apoptosis assays showed DNA fragmentation in promastigotes treated with SeNPs. In a moose experiment, prophylactic doses of biogenic SeNPs delayed the development of localized cutaneous lesions [256]. Similar in vitro data were obtained for *Crocus caspius*- and *Allium paradoxum*-mediated SeNPs [111,257].

Selenium nanoparticles are capable of exerting a dose-dependent antiparasitic effect in vitro against the Giardia deudenalis cyst, a type of parasitic flagellate protist from the genus giardia of the order diplomonads, the causative agent of human giardiasis [258]. An antihelmintic effect was shown for SeNPs from *Azadirachta indica*. *Azadirachta indica* plant extract biocompounds, enveloping selenium nanoparticles, which can combine with free proteins in the gastrointestinal tract or glycoprotein on the parasite cuticle and provoke death. Moreover, the change in worm body length after SeNP treatment may be related to these phytocompounds’ effects on the permeability of the worms’ cuticle [104].

In summary, selenium nanoparticles synthesized using biological methods are among the most promising from the point of view of further medical applications. Their properties—antimicrobial, anticancer, antioxidant, anti-inflammatory and other features—are apparently explained not only by nature itself but also by their biological environment created by “green” biofactories.

## 4. Toxicology

Toxicity is one of the most important areas of nanoparticle evaluation. Despite the wide range of useful biomedical properties, such an assessment is strictly mandatory before implementation. Most studies confirm that SeNPs are less toxic than sodium selenite. In addition, they are more bioavailable due to their small size [259]. The data presented in [259] suggest that the SeNPs’ toxicity depends on several interrelated parameters, such as the nanoparticle size, chemical composition, dose, and exposure time, affected the organism’s biological response. The toxicological studies showed that the main targets of SeNP toxicity are not only their pro-oxidant properties but also their interaction with metabolic pathways and molecular signaling pathways, including apoptosis, and the ability of small nanoparticles to penetrate various tissues. Interestingly, however, despite affecting cancer cells and causing their death, SeNPs do not harm normal cells, improve the bioenergetic phenotype of normal cells, or cause any genetic or chromatin damage [24]. Undoubtedly, the synthesis method is also important. Thus, biosynthesized SeNPs showed low toxicity in mice compared to SeNPs obtained by chemical methods, which demonstrated the important role of *Bacillus* sp. MSh-1 in the conversion of a highly toxic Se compound to the less toxic SeNPs [260]. Biosynthesized selenium nanoparticles with *A. sativum* showed interesting biocompatible features and limited cytotoxicity when compared with chemically synthesized selenium nanoparticles [115].

Little or no toxic effect is often shown toward normal cells. Thus, human dendritic cells and fibroblasts exposed to bacterial-mediated SeNPs did not show any cell viability loss, any increase in ROS release, or any significant increase in the pro-inflammatory and immunostimulating cytokine secretion (IL-12 stimulates natural killer cells and T-lymphocytes; IL-8 causes chemotaxis and activation of leukocytes; IL-6 and TNF-α cause inflammation and a systemic acute phase reaction characterized by fever). Moreover, the authors believe that inorganic selenium is unable to stimulate human DCs alone and that organic molecules on the surface of biogenic SeNPs must be responsible for the observed effect [261]. The *Kaempferia parviflora*-mediated SeNPs showed significant cytotoxicity in human gastric adenocarcinoma cells (AGS cells) but not in normal cells [218]. The selective ability to kill only cancer cells was shown for selenium nanoparticles from *Crocus sativus*, significantly reducing the growth rate of breast cancer cells (4T1 and MCF7 cells) without adversely affecting normal cells [262]. Similar data were obtained for normal cells (Vero and WI38) with SeNPs from *Penicillium verhagenii* [89]. No hazardous effect against normal Vero cells was observed for *Polycladia myrica* SeNPs [99] and CHO cells lines for *Psidium guajava* nanoparticles [138], HBL100 cell line for *Carica papaya* SeNPs [241], or THLE2 normal liver cells for *Spirulina platensis* SeNPs [95]. *Allium sativum* SeNPs did not cause damage to vertebrate erythrocytes and macrophages [115]. A lower cytotoxic effect and such reduced cytotoxicity might be due to the phytochemicals of *M. koenigii* berry extract incorporated with the SeNPs [255]. In vitro experiments with pig jejunum epithelial cells (IPEC-J2) also indicated the significant cytoprotection of *B. paralicheniformis* SeNPs against oxidative stress caused by hydrogen peroxide, as evidenced by the suppression of ROS production [79]. Such a difference in cytotoxic effects on cancer cell lines at low concentrations compared to normal cell lines may be associated with the normal redox balance of healthy cells [92].

In vivo experiments have demonstrated similar data. A significant toxic effect of SeNPs on the Wistar albino rat animal model was not found [263]. The highest doses of SeNPs led to a decrease in weight gain, a decrease in ALT activity, and an increase in blood glucose concentration compared to the control group. However, no significant changes in the antioxidant status of rats were observed. Only SOD activity in the liver was determined; therefore, the short-term intake of SeNP supplements may potentially be safe and useful from the point of view of Se deficiency or specific treatment [263]. Less acute oral toxicity in mice in comparison to SeO_2_ for SeNPs prepared with *Cleistocalyx operculatus* extract was discovered. Mice showed normal food intake and activity after consuming SeNPs during the testing period, indicating the safety of green SeNPs for humans and animals [264]. The toxic effect of selenium nanoparticles is widely studied on shrimps (a marine crustacean). The absence of toxicity was shown for shrimp hemocytes when studying the effects of nanoparticles coated with an aqueous extract of *Sargassum angustifolium* [100], on larvae of *Artemia salina* by *Enterococcus faecium* SeNPs [265], and on *B. licheniformis* nanoparticles [169]. SeNPs had a minor toxic effect at all tested concentrations for 24 h, whereas prolonged exposure could induce a toxic effect [255]. At the same time, in model toxicity zebrafish experiments, SeNPs induced malformations in the *Danio* embryos depending on the concentration. Selenium nanoparticles at a concentration of 15–25 µg/mL caused pericardial edema, tail malformation, and a decrease in heart rate in zebrafish embryos. However, lower concentration treatment did not lead to a change in heart rate or the manifestation of any cardiac abnormalities [152]. Similar data were discovered in another study with *Danio rerio*: SeNPs showed a toxic effect on embryogenesis, and the embryos’ death was detected only at high doses, but lower concentrations did not lead to a lethal effect [130]. SeNPs had no obvious toxic effects on rats, and could be used as potential candidates for cancer chemoprevention, although doses greater than 2.0 mg Se/kg-bw induced chronic toxicity (lesions in the liver, kidneys, lungs, and thymus, and apoptotic liver cells) [266]. In addition, the presence of the organic layer (phenolic compounds and flavonoids) in papaya fruit extract as an SeNP capping agent can have a significant effect on SeNPs toxicity [130]. These data are of particular importance in determining optimal concentration, and can be used in the future for therapeutic purposes.

## 5. Conclusions

Thus, strengthening SeNPs’ properties with biocompounds possessing medical potential can become an important milestone on the way to producing environmentally friendly and safe medicines for many diseases [267,268]. Each of the biosynthesis options has its own undeniable advantages. The cultivation of bacteria and fungi to produce SeNPs allows for quickly scaling the process; at the same time, synthesis with plants opens up the widest possibilities for using their own therapeutic functions, coupled with toxicity reduction. The crucial role of capping agents has been shown in much research. These molecules mainly modulate the chemical surface composition, morphology, and size distribution of nanoparticles, preventing agglomeration and enhancing the kinetics of nanoparticle reduction by forming complex structures with metal ions in precursor salts. They are capable of performing a stabilizing function. For example, it was shown that the SeNP surface was strongly adsorbed and passivated by sulfated *Ganoderma lucidum* polysaccharide molecules, leading to the formation of a homogeneous and spherical morphology. Polysaccharide molecules can be closely absorbed around the Se particles, according to the surface absorption effect of the nanoparticles, and stability may be related to the electrostatic repulsion of negatively charged sulfate groups of the polysaccharide [269]. Polysaccharide–protein complexes isolated from Asian clam (*Corbicula fluminea*) and Pu-Erh tea crude polysaccharides were stabilizers of SeNPs [228,270]. In addition, there is evidence regarding bacterial SeNPs that some biomolecules are bound more strongly than others to the core metalloid matrix, so that the diverse capping layer components differentially contribute to the overall structural characteristics of the nanoparticles [271]. Moreover, *Anoectochilus burmannicus* extract can acts as a cryoprotectant and/or lyoprotectant during the freeze-drying process of the SeNPs, resulting in the complete resuspension of the particles with the preservation of both physical and biological properties [230]. There are many such examples, not to mention the fact that most researchers approve the cell extract biomolecules’ participation as enhancers of the selenium nanoparticles biological functions. Biological molecules coating the nanoparticles not only reduce their side effects but also improve their characteristics like long-term stability and biocompatibility (speed up the uptake and retention of nanoparticles), and enhance their antimicrobial and anticancer activity [272].

The SeNPs’ antibacterial activity against well-known pathogenic microorganisms is intensively studied. The results of the antibiofilm formation are particularly important in this regard. For example, selenium nanoparticles inhibit *S. aureus* adherence and microcolony formation on polystyrene, glass, and catheter surfaces [273]. This useful property can be applied in the fight against hospital infections. Selenium nanoparticles may be considered antibacterial drug carriers. For example, one of the most exciting potential benefits of the simultaneous nanoparticles and drug application is to facilitate drug penetration into the fungi cell membrane destroyed by nanoparticles, helping against fungal infections.

One particular SeNP role involves its anticancer activity. They often demonstrate selective effectiveness in relation to cancer cells without significantly affecting healthy cells. In addition, capping agents with antitumor potential can enhance the anticancer effect; therefore, the creation of conjugates of nanoparticles and drugs of natural origin may become an important direction in this area. Thus, for instance, selenium nanoparticles decorated with troxerutin (troxerutin, commonly known as vitamin P4, which is a bioflavonoid widely present in tea, coffee, and cereals, as well as in many vegetables and fruits, and has antioxidant, anti-inflammatory, antidiabetic, and antitumor properties) were effective against breast cancer in vitro, leading to the activation of the caspase cascade pathway and invoking apoptosis by antiapoptotic proteins and gene suppression, and the enhancement of pro-apoptotic proteins and genes. Also, in vivo investigations like histopathology, hematology, and biochemical parameters revealed that the SeNPs’ significantly lowered the tumor volume of treated Balb/C mice without having any significant systemic toxicity [274]. Another tendency in achieving anticancer effects may be their protective role when simultaneously used with already known chemical drugs against cancer. Thus, SeNPs produced by the endophytic fungus *Fusarium oxysporum* conjugated with DOX (doxorubicin) can alleviate the side effects caused by DOX: the DOX–SeNP conjugate reduced ROS/RNS, 8-OHdG, and MDA levels in the liver, kidney, and heart tissues. It also restored the antioxidant enzymes and cytoarchitecture of the examined tissues, reduced genotoxicity, and increased the Bcl-2 levels; DOX reduced the number of cardioprotective metabolites, which DOX-conjugated SeNPs restored [275]. The protective effect of *Pleurotus tuber*—a regium polysaccharide—protein complex functionalized SeNPs against acetaminophen (APAP)-induced oxidative injury in HepG2 cells and C57BL/6J mouse liver cells was found [276]. SeNPs mediate redox regulation to improve antioxidant status, which, in turn, endows hepatocytes with more powerful resistance to APAP-induced toxicity [276]. SeNPs significantly improved serum T, sperm quality, and spermatogenesis and reduced Cisplatin-induced free radical toxic stress and spermatic DNA damage. It may be useful to prevent Cisplatin-induced gonadotoxicity through its antioxidant potential in Wistar rats [277].

The assortments of positive effects of SeNPs are growing from year to year. Prodigiosin-synthesized SeNPs can potentially become an antiepileptic drug. Such SeNPs delayed the onset of epileptic seizures and significantly shortened their duration. Moreover, SeNPs prevented hippocampal cell loss, oxidative stress, and neuroinflammation, restored the balance between excitatory and inhibitory neuromediators, and in particular, normalized monoaminergic and cholinergic transmission, indicating that prodigiosin SeNPs can serve as a natural anticonvulsant due to its active antioxidant, anti-inflammatory, antiapoptotic, and neuromodulating properties [278]. *Syzygium aromaticum*-mediated SeNPs significantly restored proinflammatory cytokines (interleukin-1ß, interleukin-6 and tumor necrosis factor-α) to their normal levels and suppressed the activity of glial fibrillar acid protein, demonstrating their inhibitory effect on the inflammation associated with epilepsy [245]. Green-synthesized selenium nanoparticles using *Spermacoce hispida* as a carrier of s-allyl glutathione (a glutathione analogue) attenuated the APAP-toxicity-induced elevation of kidney and liver injury markers in the blood circulation. Histological observation showed that NP pretreatment protected the morphology of liver and kidney tissue. SeNPs protected the liver and kidneys from APAP toxicity by reducing oxidative stress, enhancing the action of endogenous antioxidants, and protecting mitochondrial functions [279]. Lycopene-biosynthesized SeNPs had the best nephroprotective profile against acute kidney injury in rats via antioxidant, anti-inflammatory, antiapoptotic, and antinecroptotic activity [280]. SeNPs produced by rGSH and bovine serum albumin inhibited various inflammation and proliferation mediated pathways and could be an ideal candidate for psoriasis therapy [281]. Selenium nanoparticles functionalized with Resveratrol (RSV is a natural polyphenolic phytoalexin, found in many plants, including peanuts, eucalyptus, blueberries, cranberries, and grapes, can have antioxidant, anti-inflammatory, neurodegenerative, and neuroprotective effects, neutralizing amyloid beta peptide aggregation and its oxidative effects) maximize the RSV therapeutic potential against Alzheimer’s disease not only due to their antioxidant but also anti-inflammatory action, improving neurocognitive function and modulating signaling pathways [282]. The wound-healing activity of SeNPs from Streptomyces minutiscleroticus was studied on a model of an excised wound in Wistar albino rats. The SeNPs’ wound healing effectiveness was evaluated by in vitro methods: treatment with various SeNP doses together with an ointment base led to the healing of artificial wounds within 18–21 days, approximately equal to healing when using the standard antibiotic Lyramycin [170]. Having anti-inflammatory and antifibrotic effects, and a remarkable immunomodulatory effect, making them a suitable option for dietary supplements with a much lower toxicity risk, as well as direct antiviral properties, SeNPs can reveal new opportunities as suitable agents for this dangerous disease therapy and other viral infections with high mortality. They could be administered as an aerosol spray, a direct injection, or as thin-film transdermal patches to reduce the spread [283].

Thus, in the near future, selenium nanoparticles may open a new chapter in medical practice. The advantage of biological methods is obvious because they generate biocompatible, biologically active, and low-toxicity nanoparticles with a wide range of useful properties.

## Figures and Tables

**Table 2:** Antibacterial parameters of SeNPs.

Biosource	Strain	Activity	Standard Drug	Reference
*Lactobacillus acidophilus*	*S. aureus*	MIC_90_ = 1.2 μg/mL	Gentamycin	[67]
	*E. coli*	MIC_90_ = 9.4 μg/mL		
	*K. pneumoniae*	MIC_90_ = 6.5 μg/mL		
*Ralstonia eutropha*	*S. pyogenes*	IC = 250 μg/mL	Ampicillin	[75]
*Penicillium crustosum*	*P. aeruginosa*	MIC = 25 μg mL^−1^		[83]
*Penicillium verhagenii*	*B. subtilis*	MIC = 50 μg mL^−1^		[92]
*Spirulina platensis*	*S. typhi*	MIC = 70 μg/mL		[95]
*Sargassum angustifolium*	*Vibrio harveyi*	MIC = 200 μg/mL		[100]
*Elaeagnus indica*	*S. typhimurium*	MIC = 10 μg/mL	Ciprofloxacin	[117]
*Phyllanthus emblica*	*S. aureus*	MIC = 32 μg mL^−1^	Ampicillin	[137]
*Orange Peel Waste*	*E. coli*	MIC = 50 μg/mL	Ciprofloxacin and Gentamycin	[139]
*Azadirachta indica*	*B. cereus*	IC = 40 μg/mL	Ampicillin	[141]
*Stevia rebaudiana*	*E. faecalis*	ZOI = 11.2 mm		[180]
	*S. mutans*	ZOI = 16.9 mm		
*Saccharomyces cerevisiae*	*S. aureus*	MIC = 31.25 μg/mL	Streptomycin	[188]
*Allium sativum*	*E. coli*	ZOI = 29 mm		[189]
	*S. typhi*	ZOI = 27 mm		

**Table 3:** Antifungal activity of SeNPs.

Biosource	Strain	Activity	Standard Drug	Reference
*Calendula officinalis*	*Fusarium oxysporum*	MIC = 0.25 mg/mL and above	Cycloheximide	[107]
*Hibiscus esculentus*	*C. albicans*	MIC = 138.75 μg/mL	Ciprofloxacin	[124]
*Carica papaya*	*Aspergillus ochraceus*	MIC = 16.22μg/mL		[130]
*Capparis decidua*	*C. albicans*	ZOI = 20–30 mm		[186]
*Cinnamon oil*	*C. albicans*	IC = 0.4 mg/mL		[187]
*Urtica dioica*	*A. fumigatus*	MIC = 15.62 μg mL^−1^		[195]
	*A. niger*	MIC = 31.25 μg mL^−1^		
	*A. flavus*	MIC = 7.81 μg mL^−1^		
*Artemisia dracunculus*	*A. niger*	ZOI = 17–36 mm		[196]
	*P. chrysogenum*	ZOI = 9–14 mm		
